# Delivery strategies for malaria vaccination in areas with seasonal malaria transmission

**DOI:** 10.1136/bmjgh-2023-011838

**Published:** 2023-05-05

**Authors:** Jane Grant, Halimatou Diawara, Seydou Traore, Fatoumata Koita, Jessica Myers, Issaka Sagara, Daniel Chandramohan, Alassane Dicko, Brian Greenwood, Jayne Webster

**Affiliations:** 1 Faculty of Infectious and Tropical Diseases, London School of Hygiene and Tropical Medicine, London, UK; 2 Malaria Research and Training Center (MRTC), Universite des Sciences des Techniques et des Technologies de Bamako, Bamako, Mali

**Keywords:** malaria, vaccines, qualitative study, child health

## Abstract

**Background:**

Seasonal vaccination with the RTS, S/AS01_E_ malaria vaccine given alongside seasonal malaria chemoprevention (SMC) substantially reduces malaria in young children. The WHO has recommended the use of RTS, S/AS01_E_, including seasonal vaccination, in areas with seasonal malaria transmission. This study aimed to identify potential strategies to deliver RTS, S/AS01_E_, and assess the considerations and recommendations for delivery of seasonal malaria vaccination in Mali, a country with highly seasonal malaria.

**Methods:**

Potential delivery strategies for RTS, S/AS01_E_ in areas with seasonal malaria were identified through a series of high level discussions with the RTS, S/AS01_E_ plus SMC trial investigators, international and national immunisation and malaria experts, and through the development of a theory of change. These were explored through qualitative in-depth interviews with 108 participants, including national-level, regional-level and district-level malaria and immunisation programme managers, health workers, caregivers of children under 5 years of age, and community stakeholders. A national-level workshop was held to confirm the qualitative findings and work towards consensus on an appropriate strategy.

**Results:**

Four delivery strategies were identified: age-based vaccination delivered via the Essential Programme on Immunisation (EPI); seasonal vaccination via EPI mass vaccination campaigns (MVCs); a combination of age-based priming vaccination doses delivered via the EPI clinics and seasonal booster doses delivered via MVCs; and a combination of age-based priming vaccination doses and seasonal booster doses, all delivered via the EPI clinics, which was the preferred strategy for delivery of RTS, S/AS01_E_ in Mali identified during the national workshop. Participants recommended that supportive interventions, including communications and mobilisation, would be needed for this strategy to achieve required coverage.

**Conclusions:**

Four delivery strategies were identified for administration of RTS, S/AS01_E_ alongside SMC in countries with seasonal malaria transmission. Components of these delivery strategies were defined as the vaccination schedule, and the delivery system(s) plus the supportive interventions needed for the strategies to be effective. Further implementation research and evaluation is needed to explore how, where, when and what effective coverage is achievable via these new strategies and their supportive interventions.

**WHAT IS ALREADY KNOWN ON THIS TOPIC**

- Seasonal vaccination with the RTS, S/AS01_E_ malaria vaccine given alongside seasonal malaria chemoprevention reduces malaria in children substantially and has been recommended by WHO for use in areas with seasonal malaria transmission.
- The RTS, S/AS01_E_ vaccine has been implemented only in non-seasonal areas through an age-based strategy delivered through the Essential Programme on Immunisation (EPI) and only up to 2 years of age.
- New approaches may be required for the delivery of RTS, S/AS01_E_ in areas with seasonal malaria transmission. No other routine childhood vaccines are currently delivered following a seasonal schedule or beyond 2 years of age in these countries.

**WHAT THIS STUDY ADDS**

- This study expands current thinking by identifying four possible delivery strategies for the delivery of RTS, S/AS01_E_ in areas with seasonal malaria transmission, defines the components of a delivery strategy, and considers both age-based and seasonal vaccination strategies and their delivery systems.
- This study presents the national considerations and reasoning in determining a preferred delivery strategy, which in Mali was age-based priming doses and annual seasonal booster doses, all delivered via the routine EPI.
- Supportive interventions were identified that will be needed to increase the effectiveness of the strategies in Mali.

**HOW THIS STUDY MIGHT AFFECT RESEARCH, PRACTICE OR POLICY**

- Policy-makers and implementers can use the proposed delivery strategies and findings presented in this study, alongside other research and practical, economic and contextual considerations, to make decisions on the delivery of RTS, S/AS01_E_ in areas with seasonal malaria transmission.
- Implementation research and programme evaluation is needed on these new delivery strategies and their supportive interventions within clearly defined contexts to maintain the impressive impact achieved with seasonal vaccination in trial conditions.

## Background

Seasonal malaria chemoprevention (SMC), the monthly administration of antimalarials to children under 5 years of age during the malaria transmission season, is an effective way of preventing malaria in young children in areas with seasonal malaria, and is now being widely deployed.^1 2^ Nevertheless, malaria remains the most frequent cause of death and hospital admissions in children under 5 years of age in many seasonal areas.^3^ In 2021, WHO recommended the widespread use of RTS, S/AS01_E_ malaria vaccine in areas of moderate to high malaria transmission, including the potential for countries with seasonal malaria transmission to provide the vaccine seasonally.^4^

In the Malaria Vaccine Implementation Programme (MVIP), which introduced RTS, S/AS01_E_ into three countries with perennial transmission in 2019, four doses of the vaccine were integrated into the country’s routine Essential Programme on Immunisation (EPI) following an age-based schedule, vaccinating children up to 2 years of age.^5^ However, delivery of seasonal malaria vaccination to children up to potentially 5 years of age requires a novel delivery approach as no other routine childhood vaccines are currently delivered following a seasonal, calendar-based schedule, and no childhood vaccines are routinely given beyond 2 years of age.

This study aimed to identify the potential strategies to deliver the RTS, S/AS01_E_ vaccine alongside SMC in areas with seasonal malaria transmission, assess stakeholders’ perceptions of these strategies and develop recommendations for implementation in Mali. The study provides a first step in the identification and development of delivery strategies for seasonal malaria vaccination, and the key considerations and recommendations for its delivery in a country with seasonal malaria.

## Methods

### Study design and components

This study had three components. First, the potential delivery strategies for RTS, S/AS01_E_ alongside SMC were identified through a series of high level discussions with the RTS, S/AS01_E_ plus SMC trial investigators, international and national immunisation and malaria experts, and through the development of a theory of change (ToC). Second, these strategies were explored in qualitative in-depth interviews (IDIs) with key stakeholders at the national, regional, district, health facility and community levels. The qualitative data collection included realist interviewing^6^ to explore what delivery strategy works for who in what circumstances to achieve effective delivery of RTS, S/AS01_E_ alongside SMC. Realist approaches are theory driven with a central tenet that interventions work based on the decisions of individuals, and that these decisions are driven by mechanisms triggered in some contexts and not in others.^7^ As the study and peripheral discussions surrounding the implementation of RTS, S/AS01_E_ progressed, these strategies were adapted. Based on the qualitative data and discussions between study investigators and global experts, a fourth delivery strategy was identified. Third, following the qualitative data collection and analysis, a workshop was held in Bamako with key stakeholders from the National Malaria Control Programme (NMCP) and EPI, and key representatives from these programmes in the study regions and districts. At the workshop, the four delivery strategies and findings from the qualitative data were presented alongside the seasonal RTS, S/AS01_E_ plus SMC trial results.^8^ These findings were discussed to work towards consensus on how to deliver RTS, S/AS01_E_ plus SMC in Mali.

### Study site

The study took place in Mali. For data collection at the district and regional levels, two districts and their respective regions were included: Ouelessebougou and Bougouni districts, which lie in the southern regions of Koulikoro and Sikasso, respectively. In addition to the data collection in two districts and their respective regions, IDIs and the workshop were held with national level stakeholders in Mali. Additionally, global-level discussions contributed to the identification of the delivery strategies, as described below. Ouelessebougou and Bougouni are semirural districts, with high levels of illiteracy, where agriculture is the main occupation. The RTS, S/AS01_E_ plus SMC trial was conducted in parts of these districts from 2017 to 2021,^8^ where malaria is highly seasonal, with most cases occurring July–November. In the study districts, regions and nationally, malaria is the primary cause of outpatient consultations, hospital admissions and deaths in children under 5 years of age.^9^ Four monthly cycles of SMC are delivered by the NMCP via door-to-door campaigns in July–October, with some parts of the country currently piloting the addition of a fifth cycle. Nine different childhood immunisations are routinely delivered by the EPI programme at health centres and outreach posts. In Mali, mass vaccination campaigns (MVCs) are also employed in response to epidemics, to introduce new vaccines, or when routine coverage is low. EPI coverage is relatively high in Mali, with an estimated 77% of children receiving DTP-3.^10^

### Identification of delivery strategies and development of ToC

Potential vaccine delivery strategies were identified at the beginning of the study in September 2021 through a series of high level discussions with the RTS, S/AS01_E_ plus SMC trial investigators, international and national immunisation and malaria experts. A ToC was used (in its capacity as an aid to programme design) to consider the fit of the strategies within a potential national programme.^11^ This included consideration of the activities that would be needed to generate the required outputs through which strategy outcomes would be achieved, and therefore, the relative benefits and challenges of the specific strategies and their components (online supplemental figure S1 and table S1). The ToC was developed using the study investigators’ experience of the implementation of similar interventions, supplemented by a review of the literature on the delivery strategies of other interventions, including routine EPI vaccines, vaccination campaigns, other vertical campaigns including SMC and nutrition campaigns, and the RTS, S/AS01_E_ pilot study.

10.1136/bmjgh-2023-011838.supp1 Supplementary data

**Figure 1:**
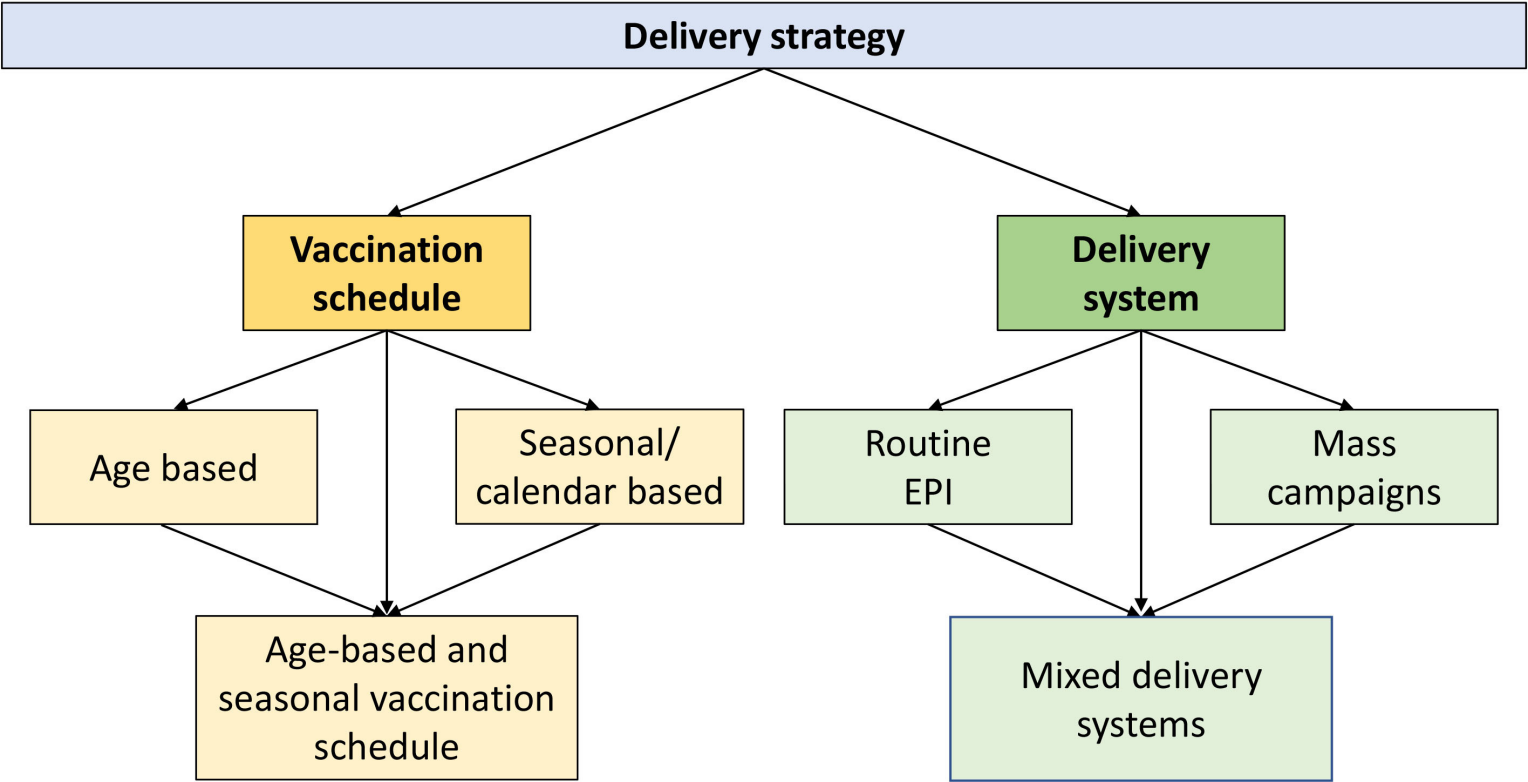
Components of the delivery strategies for RTS, S/AS01_E_ vaccination in areas with seasonal malaria. EPI, Essential Programme on Immunisation.

The vaccine delivery strategies consist of two major components: the vaccination schedule and the delivery system (figure 1). The vaccination schedule comprises the number of vaccine doses, target ages and whether vaccines are administered according to an age-based or seasonal (calendar-based) schedule. RTS, S/AS01_E_ is given as three priming injections at 1-month intervals in the first year (primary series), followed by booster doses; in the MVIP, one booster dose was given at 24 months of age, whereas in the RTS, S/AS01_E_ plus SMC trial, four annual seasonal booster doses were given until children reached 5 years of age, the age at which SMC stops. The second component is the delivery system(s) used to deliver the doses.

### Qualitative data collection and analysis

Purposive sampling was used to select: the key NMCP and EPI programme managers at the national, regional and district level; different cadres of health workers involved in the delivery of EPI vaccines and SMC; relevant stakeholders in each community; and caregivers of children under 5 years of age. The health workers were sampled from eight selected community health facilities in Bougouni and Ouelessebougou districts. These health facilities were purposively selected out of 61 health facilities in the districts to include variation across health facilities, including whether they were/were not within the RTS, S/AS01_E_ plus SMC trial sites, were situated in an urban/rural setting, and health facilities with relatively higher and lower EPI coverages. At each health facility, the health workers who worked on the EPI and SMC programmes, including the facility director, were selected, with the aim of selecting around four health workers per health facility. Caregivers were sampled from the catchment areas of the eight selected health facilities, and were selected purposively to include variation in sex, distance from the health facility and literacy. Both trial and non-trial caregivers were included to capture the perspectives of those who had/had not received the RTS, S/AS01_E_ (or control) vaccine, and to prevent against any biases derived from inclusion in the trial.

Discussion guides for the IDIs were developed based on the delivery strategies and ToC. Additionally, several theoretical frameworks were drawn on to frame the questions probing participants’ perceptions of the different delivery strategies including the WHO health systems building blocks,^12^ Roger’s Diffusion of Innovations theory^13^ and Bowen’s feasibility framework.^14^

Different discussion guides were used for health programme managers, health workers, caregivers and community stakeholders. At the beginning of the interviews, the background to the seasonal RTS, S/AS01_E_ plus SMC trial was described, and the results of the trial were presented using a graphic (online supplemental figure S2). The delivery strategies were then presented by the interviewers to the respondent, either using figures or verbally. After this, the interviewer asked a series of scripted, open-ended and probing questions to facilitate discussion on each strategy. Finally, the participant was asked to compare the strategies and give their overall preference and rationale for which strategy should be used to deliver RTS, S/AS01_E_. The level of detail in which the trial results and delivery strategies were presented and discussed varied according to the participant group.

**Figure 2:**
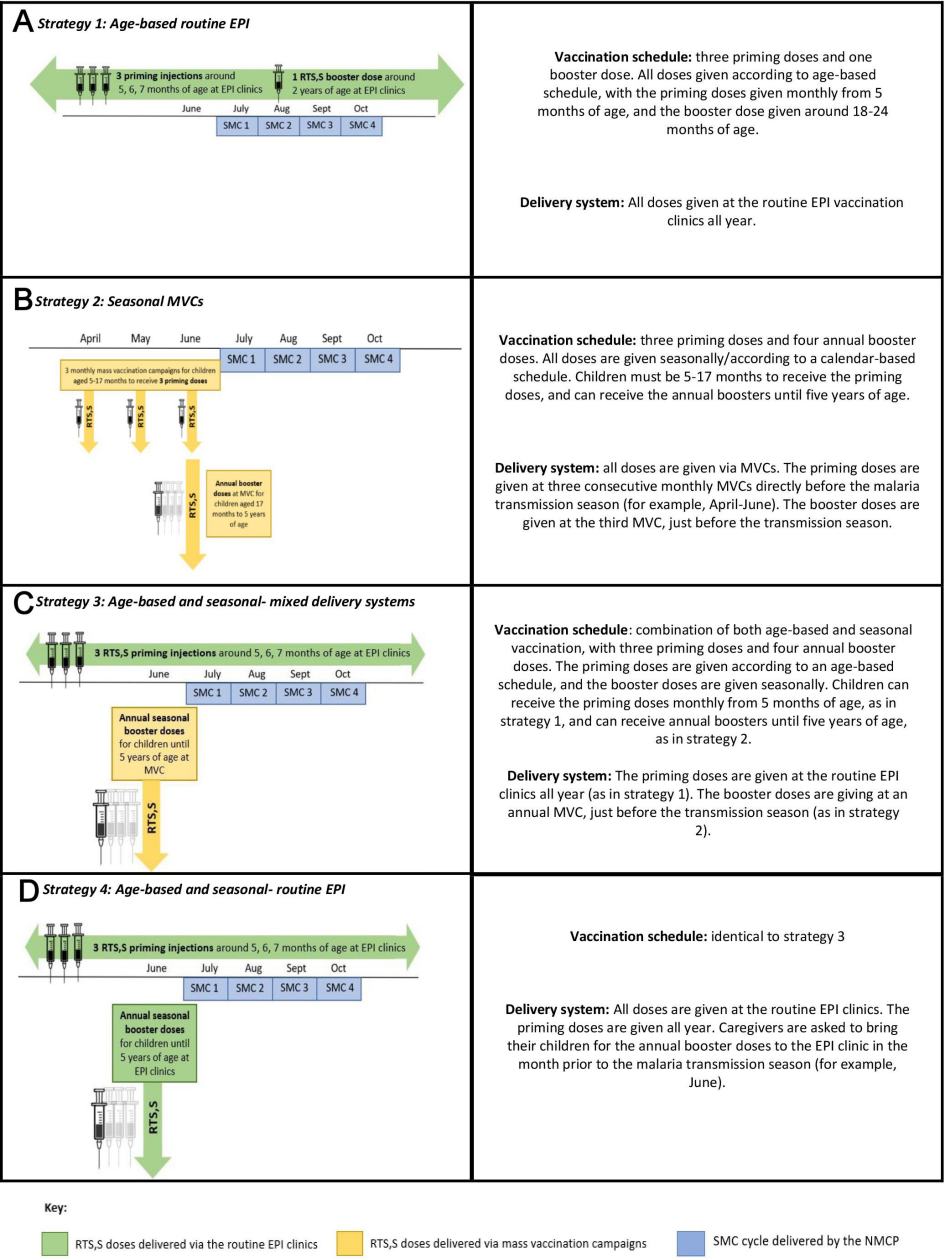
Potential strategies for the delivery of RTS, S/AS01_E_ alongside SMC. EPI, Essential Programme on Immunisation; MVCs, mass vaccination campaigns; NMCP, National Malaria Control Programme; SMC, seasonal malaria chemoprevention.

Led by the discussion guides, the interviewers interrogated the context in which RTS, S/AS01_E_ and SMC would be delivered, and how this affected participants’ perceptions and recommendations for the delivery. This interrogation was supported by presentation and discussion/validation of context-mechanism-outcome (CMO) configurations that represent theories on the factors (contexts) and mechanisms that lead to the recommendations for the delivery of the interventions. The CMOs were developed from the ToC and from reviewing preliminary data from field notes made during the programme manager IDIs.

Interviews were conducted in French and Bambara by four trained Malaria Research and Training Centre researchers. All interviews were digitally recorded. During the IDIs, a second researcher took field notes of the main points and key observations from the interview. These were used to review the emerging key points from the data and improve the interview process. The interviews in French were transcribed verbatim and the interviews in Bambara were simultaneously transcribed and translated into French. All transcripts were subsequently translated into English, and imported into NVivo for coding and analysis. Transcripts were anonymised but the interview number and participant group were retained to assist the analysis. The transcripts were coded by two of the London School of Hygiene and Tropical Medicine (LSHTM) study researchers using a framework analysis approach, with an initial coding framework developed based on the key themes from the interview guides.^15^ These themes were then populated inductively with subthemes as they were identified from the data. During the analysis, detailed notes were recorded by the two coders to inform the interpretation of the results. The coding and the synthesised results were discussed among the researchers at LSHTM and the Malaria Research and Training Centre at multiple points during the analysis to help verify the coding and ensure the credibility and confirmability of the findings.

The Standards for Reporting Qualitative Research was used to ensure rigorous reporting of the qualitative results (online supplemental table S2).

### National stakeholder workshop

A workshop was held in Bamako in July 2022 with key stakeholders from the NMCP and EPI, and key representatives from these programmes in the study regions and districts. At the workshop, the four delivery strategies and themes from the qualitative data were presented, alongside the 5-year efficacy and safety results from the seasonal RTS, S/AS01_E_ plus SMC trial.^8^ The findings of the qualitative study including the original three delivery strategies, together with the newly identified fourth delivery strategy, were used to discuss and work towards consensus among the stakeholders on how to deliver RTS, S/AS01_E_ plus SMC in Mali. In addition, through the presentation and discussion of the qualitative findings, the workshop helped to validate the findings and interpretation of the data. Minutes were taken to record the workshop.

### Patient and public involvement

The views and experiences of caregivers, community stakeholders, and health workers were sought as participants in this study, and these groups were not involved in the design, conduct, reporting or dissemination plans of this study. Malaria and immunisation programme managers in Mali contributed to the research question, study design and dissemination. An author reflexivity statement is provided in online supplemental appendix.

## Results

### Delivery strategies for RTS, S/AS01_E_ alongside SMC

The first three delivery strategies (1–3) identified at the beginning of the study, and discussed during the IDIs, were age-based routine EPI (strategy 1), seasonal MVCs (strategy 2) and age-based and seasonal-mixed delivery systems (strategy 3) (figure 2). From the perceptions of the three strategies discussed during the IDIs, and discussions between the trial investigators and global experts, it emerged that implementing MVCs brought large feasibility challenges, but there was strong interest in implementing seasonal booster doses. Therefore, a new strategy, age-based and seasonal-based routine EPI (strategy 4), was developed (figure 2D).

In all four strategies, SMC is given as usual by the NMCP via four, monthly campaigns during the malaria transmission season. In each of the strategies, children can receive the first priming dose of RTS, S/AS01_E_ from 5 months of age, and with a minimum of 4 weeks between doses. In strategies 2–4, there is an interval of 12 months between the booster doses.

### Perceptions of the delivery strategies and recommendations for delivery

**Figure 3:**
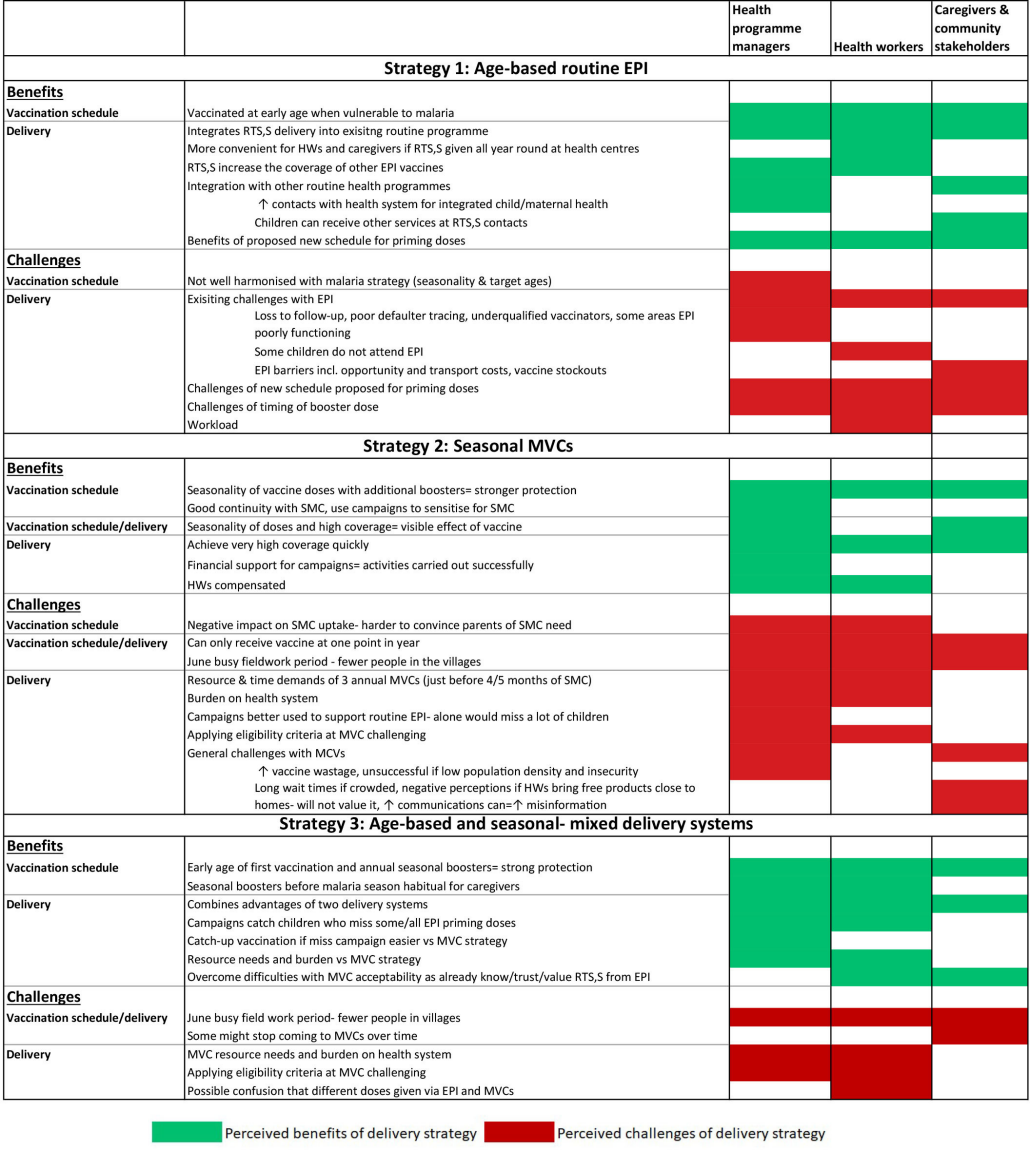
The benefits and challenges are labelled as to whether within the strategy, they relate to the vaccination schedule, or the delivery system(s) used. EPI, Essential Programme on Immunisation; HWs, health workers; MVCs, mass vaccination campaigns; SMC, seasonal malaria chemoprevention.

One hundred and eight participants were interviewed (table 1). The results are presented according to the three major participant groups: programme managers, health workers and the community-level participants (caregivers and community stakeholders) (figure 3). Perceptions of the benefits and challenges of the strategies, and recommendations for delivery, were similar across participants from the RTS, S/AS01_E_ + SMC trial and non-trial sites.

**Table 1:** Participants in the in-depth interviews

Type of participant	Total
EPI and malaria programme managers	
National level	8
Regional level	7
District level	10
Health workers	32
Caregivers of children enrolled in the RTS, S/AS01_E_+SMC trial	17
Caregivers of children under 5 years of age not enrolled in the trial	26
Community stakeholders*	8
Total	108

*Chiefs, local health association members, women’s group leaders, youth group leaders and farmers association leader.

EPI, Essential Programme on Immunisation; SMC, seasonal malaria chemoprevention.

Benefits and challenges of the three proposed delivery strategies (1–3) according to the participant groups (figure 3).

### Strategy 1: age-based routine EPI

A major benefit of this strategy described by all groups is that it fully integrates the delivery of RTS, S/AS01_E_ into an existing routine programme, taking advantage of the EPI infrastructure and resources, making the strategy cheaper and more sustainable. Participants also stated that health workers and communities already know and are used to EPI, and many children are reached through it. Additionally, programme managers and health workers discussed how they have previously successfully introduced new vaccines into the programme. The caregivers and community stakeholders felt that this strategy would be relatively easy to implement, given that the EPI is already a habit for most caregivers, and is well accepted and trusted, with EPI vaccines valued and considered effective having successfully reduced diseases such as measles. They believed that if RTS, S/AS01_E_ is delivered through this trusted programme, it would give the new vaccine credibility and reduce negative rumours. Programme managers and health workers also hoped that introducing the vaccine into the EPI would have a beneficial effect on the overall programme due to the burden of malaria in these communities, and the demand for a malaria vaccine, and that the new RTS, S/AS01_E_ contacts would provide new opportunities to catch-up other missed EPI vaccines, increasing EPI coverage.

All groups also discussed the benefits of having the three priming doses given alone at new vaccination contacts. The programme managers and health workers appreciated that this would make the EPI programme more continuous and easier for caregivers to remember, with children coming almost every month from their first year of life. Health workers added that there are many EPI vaccines in the first 3 months of life, and it is beneficial to add RTS, S after a short break from this busy period. Many caregivers preferred to receive RTS, S/AS01_E_ alone at new contacts due to concerns that when multiple vaccines are given together, the side effects worsen. Furthermore, if given at new contacts, more people would be aware that their child is receiving the malaria vaccine, rather than just multiple ‘EPI vaccines’. Health workers preferred new vaccines to be given alone to more easily monitor their side effects.

Participants emphasised the challenges in adding new EPI contacts, especially booster doses. For the primary series, programme managers and health workers stated that it would be difficult to get caregivers to bring their children to the clinic for the new contacts, as there is already significant loss to follow-up in the programme and many children do not come between 3 and 9 months, when they return for measles vaccination (MCV). Some caregivers and community stakeholders were concerned that some would think this is too many contacts and vaccines, given the existing barriers to attending EPI. Participants also stated that it would be very challenging to get older children to return for the fourth dose after the long gap from the third dose and with many caregivers perceiving EPI ending at 9 months of age. At this time point, many caregivers may not remember the need to return to the clinic and may have lost their vaccination card. Participants suggested that additional activities would be needed to establish the new contacts, especially the booster dose, including intensive sensitisation and mobilisations with strong involvement of community health volunteers, systematic reminders, tracing the defaulters and possibly providing other motivators, such as bed nets. Some participants suggested that, if possible, the primary series be given at existing contacts and the age of the booster dose lowered, and given in combination with MCV-2 at 15 months. Health workers also expressed concerns about their increase in workload if this strategy is adopted, including undertaking additional activities necessary to ensure caregivers attend all required contacts.

### Strategy 2: seasonal MVCs

A major benefit discussed by participants was the ability of MVCs to achieve high coverage very quickly; the more accessible vaccination sites closer to communities, alongside the intensive mobilisations and communications that accompany campaigns, were predicted to reach many children, including those who would not attend EPI. Caregivers and community stakeholders explained that the intensive communications are motivational, resulting in high awareness. Caregivers would feel motivated and gain confidence by seeing others take their children to the campaign for a new vaccine. The high coverage, alongside the strong protection expected from the four seasonal booster doses, would result in a visible protective effect of the vaccine, maintaining high coverage of the later doses.

Despite these benefits, there were major concerns about this strategy, in particular the required resources and impact of delivering three annual MVCs, immediately before 4 or 5 monthly SMC campaigns. The large demands on financial and human resources for MVCs was considered a major challenge by programme managers, with reports that some districts already struggle to mobilise resources to deliver 4 monthly SMC campaigns. Additionally, national programme managers discussed how the campaigns would have to be funded by partners and that they preferred not to be financially dependent on partners who will not support them indefinitely. In addition, when multiple partners support a programme in different areas, such as for SMC, the programme lacks cohesion. Furthermore, programme managers and health workers expressed concerns about the burden that these intensive 7 or 8 months of malaria campaigns would place on the workload of health workers and parents and on the functioning of the broader health system. Health workers noted that health centres and EPI clinics are often empty during campaign days. However, health workers suggested that the negative impacts of campaigns could be minimised if well organised with additional qualified health workers hired. Furthermore, the campaign platform could be used to deliver other interventions, such as MCV-2.

While participants appreciated the value of the seasonal vaccine delivery, all groups expressed concerns about the ability of children to receive the vaccine only at one time point in the year. Participants did not like that some children would have to wait until they were the eligible age at the time of the campaign to receive their first dose, and that those who were just under the eligible age to receive the primary series, or who missed the campaign, would be unprotected during the transmission season, particularly as June is a busy period for field work. Almost all programme managers and health workers thought that catch-up vaccination would be needed, either through the routine EPI programme and tracing with referral of children who did not attend the campaign, or by fixed-site distribution alongside SMC resulting in additional costs and operational difficulties.

Programme managers and health workers felt that it would be difficult to determine age eligibility for the primary series during the MVC, and whether children had received the priming doses necessary to be eligible to receive a booster dose. This was seen as a big obstacle as many caregivers would lose or not bring their vaccination cards; health workers are very busy during MVCs and do not have time to check registers; they are pushed by caregivers to vaccinate ineligible children. National programme managers stated this is particularly difficult in areas with internally displaced persons, as they often do not have their vaccination cards and are not on registers.

### Strategy 3: age-based and seasonal-mixed delivery systems

This strategy was seen to combine the main advantages of the two strategies described above, in terms of vaccination schedules and delivery systems. In this strategy, infants receive primary series early in life through the EPI when they reach the age eligibility. Caregivers appreciate receiving the primary series via EPI when their children are young and they are used to attending EPI. Then, children receive efficacious seasonal boosters, potentially until 5 years of age, delivered via MVCs thus achieving high coverage for these doses. Many caregivers liked the idea that later doses would be given closer to home via well-advertised MVCs, as it would be potentially difficult taking older children to the EPI clinic as by this time point they might forget that this is required; additionally, many mothers have younger children at this time point, focussing more on their health. Some caregivers also mentioned that it would be physically difficult taking multiple children to the EPI clinic.

Additional advantages of this strategy were that the booster campaigns could be used to catch children who missed some or all of the routine EPI priming doses but are still within the allowed age, increasing the coverage of these doses. Furthermore, for children who miss the booster campaign, it would be possible to provide catch-up vaccination at routine EPI clinics which will be holding vaccine for primary vaccination. Programme managers suggested that these children could be identified by using the registers with the help of community health volunteers, or by checking vaccination cards during SMC distribution.

The main challenge discussed for this strategy was the need for additional resources and the burden on the health system of adding even one annual MVC, which would result in 5–6 consecutive months of malaria campaigns every year. Additional challenges perceived by health workers were that some caregivers would be confused by the same vaccine being given via both the routine EPI and MVC and by the need to come to both for different doses.

### Recommendations on strategies 1–3 from the IDIs

While similar challenges and benefits were discussed by all categories of participants, the final recommendations for which of the three strategies should be used to deliver RTS, S/AS01_E_ alongside SMC, and the rationale for this decision, varied between participant groups, including between different levels of programme managers (table 2).

**Table 2:** Delivery strategy recommended by each participant group during IDIs and from the national workshop, and rationale for the recommendation

	Main strategy* recommended and rationale for recommendation	
National programme managers	Strategy 3: age-based and seasonal-mixed delivery systems	Primary series in routine system sustainable and RTS, S improve EPI coverage ↑ efficacious seasonal boosters with ↑ coverage from campaigns Campaigns catch those who missed EPI
Regional and district programme managers	Strategy 1: age-based routine EPI	Integrates with current system and hence cheaper Less burden on health system Less reliance on partners More sustainable
Health workers (HWs)	Strategy 1: age-based routine EPI	Easier as existing programme HWs/caregivers are already used to it Cheaper
Caregivers and community stakeholders	Strategy 3: age-based and seasonal-mixed delivery systems	Early protection when used to EPI Yearly seasonal protection from boosters when children older Campaigns closer to home and easier for older children
National workshop*	Strategy 4: age-based and seasonal-based routine EPI	Integrates into current system=cheaper and ↑ sustainable ↑ efficacious seasonal boosters

*Strategy 4: age-based and seasonal-based routine EPI was only included in discussions at the national workshop, after the IDIs. During the IDIs, only strategies 1, 2 and 3 were considered.

EPIs, Essential Programme on Immunisation; IDIs, in-depth interviews.

The majority of national programme managers recommended age-based and seasonal-mixed delivery systems (strategy 3), as using the routine EPI for the primary series is sustainable and would improve EPI coverage, and strong, later protection would be provided from the seasonal booster doses that would have high coverage from the MVCs. Furthermore, catch-up at the seasonal MVC would increase the coverage of the primary series. The national programme managers liked that the routine EPI strategy^1^ would be more sustainable and cheaper, but they did not recommend it as it would provide less protection, being non-seasonal and providing coverage only up to 2 years of age. Despite this, the majority of regional and district programme managers recommended this strategy as it better integrates with the current system, and they had concerns about the pressures an annual MVC (strategy 3) would put on community health facilities. Programme managers at all levels stated that the MVC strategy^2^ was not feasible due to the very high costs and burden on the health system.

The recommendations from the health workers were more divided between the strategies. However, the majority recommended age-based routine EPI^1^ because of the ease and low costs of using the existing programme. The second most common recommendation was age-based and seasonal-mixed delivery systems^3^ for similar reasons to the programme managers. A few health workers recommended seasonal MVCs^2^ with its strong communication component and ease of access for more rural caregivers, but the majority found this strategy less acceptable due to the large costs and workload, and disruption of routine activities.

Some programme managers and health workers also suggested that RTS, S/AS01_E_ could be delivered first by MVCs and then fully or partially integrated into the routine EPI. This follows a common pattern of vaccine introduction in Mali and the strong mobilisations and communications that would be needed for successful MVCs would help achieve awareness and acceptance for the new vaccine, and build coverage. As the MVCs would only be employed for 1 or 2 years, the issue of sustainability would not arise, and some participants preferred using campaigns in this way to support the routine programme, rather than setting them up as parallel programmes.

The majority of caregivers and community stakeholders preferred the age-based and seasonal-mixed delivery systems strategy^3^ as caregivers liked the combination of the early and seasonal protection, and are used to EPI for young children with MVCs being an easier way of accessing the later doses. Some participants recommended the routine EPI strategy^1^ because of ease and their trust in the routine programme. Only two caregivers recommended the MVC strategy,^2^ but this group of participants did not find this strategy unacceptable, unlike the programme managers and health workers. The caregivers and stakeholders frequently stated that despite any preferences, they would find any of these strategies acceptable due to the burden of malaria and the importance of a malaria vaccine to them, but they emphasised the need for good communications and understanding for any of these strategies to work.

### Recommendations on strategies 1–4 from the national workshop

#### Strategy 4: age-based and seasonal-based routine EPI

Fifteen stakeholders attended the national workshop held in Bamako on 29 July 2022, including six representatives from the National NMCP and EPI, four from the NMCP and EPI in each of the study regions and districts and one public health researcher. After reviewing the 5 year trial efficacy results, participants felt that the four age-based doses (strategy 1) did not fit well with the evidence showing the efficacy of a seasonal seven dose strategy, vaccinating children up to 5 years of age.

Overall, workshop participants recommended that the age-based and seasonal-based routine EPI^4^ should be used to implement the vaccine. This was decided due to the feasibility issues with MVCs, and the desire to use existing systems for delivery, to reduce costs and improve sustainability. This was emphasised by the district level participants, who stated that there were too many campaigns in their districts, and were concerned that community health facilities would not be able to cope with another campaign. However, participants shared concerns that the low mobilisation in this strategy would result in poor coverage, as caregivers would need to bring children up to 5 years of age to a vaccination centre every June, which is not aligned with the current routine EPI strategy. Participants suggested that for this strategy to be effective, the communication and social mobilisation that usually accompanies a campaign could be provided in parallel with the distribution of seasonal booster doses at the health centre, taking advantage of the community health volunteers, stakeholders and organisations already in place to help deliver this. However, additional financial resources would be needed, and partners would need to commit to supporting routine communications. Additionally, some participants suggested that for the first 2 years prior to largescale implementation of RTS, S/AS01_E_, MVCs could be implemented in selected high-burden areas to create enthusiasm for the vaccine and increase accessibility for the introduction.

## Discussion

This study identified four strategies for the delivery of RTS, S/AS01_E_ alongside SMC in areas with seasonal malaria, defining the delivery strategy as the RTS, S/AS01_E_ vaccine schedule and the delivery system(s) used to deliver it. Overall, participants in the interviews and national workshop preferred the vaccination schedule in strategies 3 and 4, with the first three priming doses (primary series) given according to an age-based schedule in the first year of life, and seasonal annual booster doses. This was due to the fact that unlike the other two vaccination schedules, children are both protected early in life, and receive yearly seasonal protection past 2 or 3 years of age, as would be provided by strategy 1.^8^ However, there was discordance and discussion over how these seasonal booster doses should be delivered, as they do not fit within the current EPI strategy, both in terms of seasonality and target age group.

Despite the predicted high coverage through campaigns, the participants in this study had major concerns about the required resources and burden that adding annual seasonal malaria MVCs would have on the wider health system. Several previous studies have highlighted the negative effects that mass campaigns can have on routine health systems particularly at the district level, including the financial motivation for health workers to work on campaign instead of routine activities, the absence of health workers from health centres and the reduction or discontinuation of routine services during campaigns.^16–21^ This was emphasised by participants in the broader context of Mali where many mass campaigns are delivered regularly, including SMC, nutrition week, anthelminthic drug administration, bed net distribution and reactive and introductory MVCs. While the single annual MVC in the age-based and seasonal-mixed delivery systems strategy (strategy 3) was seen as more feasible than the three annual MVCs (strategy 2), this would still result in 5–6 months of consecutive malaria campaigns every year, which was concerning especially for district level participants. This burden of the workload associated with campaigns, particularly at the district level, is reflected in the results where the lower level programme managers and health workers overall did not recommend the strategies involving MVCs.

Due to the health system impacts of MVCs, and the perceived unsustainability of these impacts alongside the high costs and need for financing by partners, this study found an overall desire to fully integrate the delivery of RTS, S/AS01_E_ into the routine EPI system. Participants at all levels valued the routine EPI programme as a more feasible and sustainable delivery system, already known and trusted by communities. This led to the creation of a fourth strategy during the study, the age-based and seasonal-based routine EPI (strategy 4), with the age-based primary series and seasonal booster doses all delivered at the routine EPI clinics.

There were concerns about the coverage that strategy 4 would achieve. Currently, all EPI vaccines are given year-round at clinics according to an age-based schedule, vaccinating children up to 23 months of age with the last vaccine (MCV-2) scheduled at 15 months of age. Contrastingly, this strategy would require all children up to 5 years of age to come to the EPI clinic at one point in the year, before the malaria transmission season. Historically, EPI programmes have focused on children below 12 months of age, with MCV-2 recently introduced as one of the first childhood vaccines delivered beyond this age. MCV-2 has experienced significantly lower coverages than for MCV-1, partly due to continued perceptions that EPI ends after infancy, and insufficient training of health workers resulting in issues with health worker attitudes and knowledge of EPI in the second year of life.^22 23^ The coverage of MCV-2 in Mali is estimated at 33%.^10^

Supportive interventions are needed to achieve high coverage of RTS, S/AS01_E_ past the primary series in the routine EPI. Various interventions aimed at improving routine childhood immunisations have been tested, including interventions targeting communication and mobilisation, reminder/recall, incentives and provider-directed strategies.^24 25^ However, while many of these strategies have been suggested to improve coverage of immunisations, specifically in the second year of life, to our knowledge, none have been formally evaluated nor the contexts in which they are effective determined.^22 23 25–27^

The participants in this study suggested, that in the Malian context with a high burden of malaria and trust and demand in EPI vaccines, but with no experience of routine seasonal vaccination and vaccination past 15 months of age, the most effective interventions would be those involving: intensive communications and sensitisations to ensure that communities are aware of and understand the new vaccine and how to receive it; reminders to caregivers about upcoming doses; tracing non-attending children; training and supervision of health workers. One possibility for reminders and defaulter tracing would be to introduce an electronic reminder system, sending reminders for upcoming and missed vaccine contacts, which has shown some success in small pilot studies in sub-Saharan Africa.^28^ In areas with low literacy, voice Short Message Service (SMS) or phone calls can be used.^29^ This system could be combined with an electronic immunisation register to track receipt of vaccine doses at the individual level, also helping to overcome the challenges predicted in this study in relation to the retention of vaccination cards and assessment of booster dose eligibility.^30^

Participants in this study stressed that community health volunteers, who are close to and trusted by communities, should be key in delivering supportive interventions, including sensitising caregivers about upcoming vaccinations, and tracing and referring non-attending children. Participants noted that while these workers have essential roles, their participation in routine health activities has become neglected and underfunded. In Kenya, community health workers have played an important role in increasing immunisation coverage by tracing children and ensuring they do not miss or delay their immunisations.^31^ Participants also suggested that community distributors of SMC, who go door-to-door during the rainy season, could examine child vaccination cards and provide messages about RTS, S/AS01_E_ and referrals to the EPI clinic for children who did not receive their booster dose in the previous month. The referral and messaging about RTS, S/AS01_E_ during SMC contacts, and vice versa, could assist the integration of the two programmes and the understanding of the need for both interventions. It will be important to consider and assess the effect that RTS, S/AS01_E_ has on perceptions and coverage of SMC, and how this is influenced by the ways in which it is delivered.

The main advantage of MVCs discussed by all levels in this study was the strong communications and mobilisations component normally absent from routine EPI, which results in high awareness and motivation, and high coverage. Given the need for caregivers to bring their older children to EPI at one specific point in the year in the recommended age-based and seasonal-baed routine EPI strategy, workshop participants suggested the communication and social mobilisation that usually accompanies a campaign could be provided in parallel with the distribution of seasonal booster doses at the health centre, delivered by community health workers and other important community groups. However, a barrier to this would be the willingness of partners to support strengthening routine communications and programmes.

Participants in this study also raised some challenges with the delivery of the primary series in the EPI programme. There has been an assumption that it is easy to integrate new vaccines or other interventions into existing EPI programmes, but this study raised concerns about adding three new doses in an EPI schedule that is becoming increasingly crowded. While caregivers in this study often preferred RTS, S/AS01_E_ to be given alone at new contacts due to concerns over side effects and increased visibility of the vaccine, significant challenges were raised with adding new contacts into the schedule, and again, supportive interventions focusing on communication, mobilisation, reminders and recall were suggested as required for caregivers to come to these new contacts. Additionally, lessons from the RTS, S/AS01_E_ pilot study suggest that clearly thought out guidelines and strong training and supervision are needed for health workers to implement the new vaccine schedules, particularly with regard to age eligibility and what happens when children do not come when they were supposed to.^32^

While a full realist evaluation was not undertaken, the qualitative data collection in this study added realist approaches to questioning participants.^6^ The use of CMO configurations at the end of the interview were valuable in providing direct and explicit explanation of specific contexts and mechanisms leading to participants’ recommendations for the delivery strategies. For example, health workers discussed the advantage of being compensated for campaigns in the interviews. However, when this was then questioned using a CMO, it became clear that while compensation did motivate health workers to some extent, it did not lead to health workers wanting to deliver RTS, S/AS01_E_ via MVCs because of their increased burden of work and the perceived ease of delivery via the EPI. Additionally, explicitly including questions surrounding context in the interviews helped to centre the context in which the delivery of RTS, S/AS01_E_ was being considered. For example, these questions established the perceived success of, and trust in, the EPI programme in Mali, and therefore, partly why participants considered introducing the vaccine into routine EPI an easier option.

The delivery strategies identified in this study apply beyond Mali, and can be used for consideration by other countries with seasonal malaria transmission. Each country should tailor their delivery of the malaria vaccine to their specific contexts, including in terms of their profile of malaria transmission and seasonality, and the strengths of their delivery systems, in particular their EPI programme, including in areas of insecurity. Where malaria seasonality varies, some countries may choose to vary the delivery strategy used within the country. For example, Ghana is currently delivering RTS, S/AS01_E_ vaccine in parts of the country with perennial transmission using strategy 1, but if scaled up within the country, could choose to deliver seasonal booster doses of the vaccine in the parts of the country with strong seasonal malaria.^4 27^ Additionally, while this study focused on the delivery of the RTS, S/AS01_E_ vaccine, the delivery strategies identified are applicable to any malaria vaccine with similar target ages and efficacy that wanes over time (therefore, requiring regular boosters), such as the R21 vaccine.^33^

While the delivery strategies, and the definition of what a delivery strategy for RTS, S/AS01_E_ alongside SMC is, are generalisable beyond Mali, the perceived challenges and benefits of the strategies, and recommendations made in this study are specific to Mali. This study included a wide range of respondents but caregivers, health workers and district programme managers were only interviewed in two districts, and therefore, the perceptions of the strategies from these groups were specific to the context of their districts. Both study districts are semirural, and caregivers and health workers in more remote or urban communities may have different perspectives on the delivery strategies. However, broader applicability was gained by the inclusion of national programme managers and regional programme managers, who raised wider points such as how instability and weaker EPI might affect the success of the delivery strategies in other areas. Additionally, there was general agreement on the challenges and benefits of each strategy among the national, regional and district programme managers. Another limitation was that strategy 4 was only developed following the IDIs, so while it was discussed at the national workshop, it was not interogated during the interviews. This study was also limited by its prospective nature, and participants’ recommendations were preliminary and based on the currently available evidence at the time of the study (November 2021–July 2022). It is possible that the recommendations of participants would have differed had empirical evidence of the impact of the strategies been available but this is not yet the case. Further decisions on how to deliver RTS, S/AS01_E_ in Mali and other countries will need to take into account further considerations, including: the modelled efficacy of the different delivery strategies in areas of differing seasonal malaria intensities; the comparative cost-effectiveness of the strategies, especially for the choice between strategies 3 and 4; the number of vaccine doses required and available; and the financial and technical support available.

## Conclusions

Four strategies for the delivery of the RTS, S/AS01_E_ vaccine alongside SMC in countries with seasonal malaria transmission were explored. Key considerations in the development of the delivery strategies for seasonal malaria vaccination were outlined, alongside recommendations for Mali where the preferred strategy was a combination of age-based priming doses followed by seasonal booster doses, all delivered via the routine EPI programme. Supportive interventions are needed for the successful delivery of RTS, S/AS01_E_, given the novel nature and complexity of delivering vaccine doses seasonally and to an expanded age group. Further implementation research and evaluation is needed for these new strategies, including for the supportive interventions needed to increase the effectiveness of the strategies.

10.1136/bmjgh-2023-011838.supp2 Supplementary data

## Data Availability

Data are available on reasonable request. Parts of the datasets used and/or analysed are available from the corresponding author on reasonable request. The transcript files from the programme managers will not be shared in full as the participants are potentially identifiable to a person familiar with the context due to their descriptions of their roles within the immunisation and malaria programmes, and how their roles shape their responses on the delivery of the vaccine.
