# Supplementary material for: Delivery strategies for malaria vaccination in areas with seasonal malaria transmission
Source: BMJ Glob Health. 2023 May 5;8(5):e011838. doi: 10.1136/bmjgh-2023-011838 (PMC10163455; doi:10.1136/bmjgh-2023-011838)
Supplement: Supplementary data [file bmjgh-2023-011838supp001.pdf]

SUPPLEMENTAL MATERIAL

1. Author reflexivity statement
2. Figure S1 and Table S1: Theory of Change
3. Figure S2: Trial results figures
4. Table S2: Standards for Reporting Qualitative Research

Author Reflexivity Statement

1. How does this study address local research and policy priorities?

Malaria is the leading cause of outpatient consultations, hospitalisations and deaths in children in Mali. This burden remains despite the wide-scale implementation of effective preventive interventions. The goal set forth by the National Malaria Control Programme and Mali's Ministry of Health is to eradicate malaria in Mali by 2030; new approaches to malaria prevention will be needed to meet this goal. The clinical trial demonstrating the efficacy of combining seasonal malaria vaccination with seasonal malaria chemoprevention was conducted in Mali. The Mali Ministry of Health is highly interested in the potential implementation of a malaria vaccine, and will be applying to GAVI for supply of the RTS,S/AS01$_E$ vaccine. This study provides a first step in the development of the routine delivery strategies for seasonal malaria vaccination, and the key considerations and recommendations for its delivery in Mali. The national immunisation and malaria programme managers in Mali were supportive of this study and inputted to the study at multiple points.

2. How were local researchers involved in study design?

The conceptualisation of the study and development of the protocol and study materials were done jointly by the authors in Mali (HD, ST, AD, IS) and in the U.K. (JG, JW, BG, DC). All of these researchers made substantial and important contributions to the study design.

3. How has funding been used to support the local research team?

This project has supported the development of an experienced social science team at the Malaria Research and Training Centre (MRTC) in Mali. Additionally, this project supported training for two medical students from the University of Sciences, Techniques and Technologies of Bamako in qualitative research and gave them practical experience of qualitative data collection.

4. How are research staff who conducted data collection acknowledged?

The three members of the research team who significantly contributed to the data collection are authors ST, HD, FK, as stated in the contributions section. The other researchers who helped to collect the data but did not significantly contribute to shaping how the data were collected or to how study findings were interpreted are thanked in the acknowledgements section.

5. Do all members of the research partnership have access to study data?

All members of the partnership have access to the study data through JG, ST, HD and JW.

6. How was data used to develop analytical skills within the partnership?

This was not specifically addressed during the study.

7. How have research partners collaborated in interpreting study data?

Interpretation of the data was a collective effort from all authors. In-person and virtual meetings were held during data collection and analysis between HD, ST, JG, JW, JM and FK to discuss the interpretation of the study findings. The findings were presented to the other authors during the study to gain their input.

8. How were research partners supported to develop writing skills?

JG wrote the first draft of the manuscript and was guided and supported in this by JW and other senior authors. All authors reviewed the manuscript and made substantial contributions to it.

9. How will research products be shared to address local needs?

The preliminary results of the qualitative data collection were disseminated to key stakeholders in Mali during a national workshop as part of the study. The final results of the study as presented in the manuscript will be disseminated to these stakeholders. The results will be translated into French so that they can be accessible to policy makers and implementers in Mali, and other French speaking West African countries.

10. **How is the leadership, contribution and ownership of this work by LMIC researchers recognised within the authorship?**

Five out of ten of the authors are from Mali.

11. **How have early career researchers across the partnership been included within the authorship team?**

We have included early career researchers from both partner organisations (JG, ST, FK, JM) within the authorship team. The early career researchers contributed to all stages of the study, from study conception to analysis and paper-writing.

12. How has gender balance been addressed within the authorship?

Five of the authors are female (JG, FK, JM, JW, HD) and five are male (ST, IS, DC, AD, BG).

13. How has the project contributed to training of LMIC researchers?

JW, an experienced social science researcher, mentored the team throughout the study. HD and ST provided training on qualitative techniques, supervision and support to FK and the other data collectors. ST was supported by HD, AD, JW and JG to gain experience as a social science researcher and to advance in this field.

14. How has the project contributed to improvements in local infrastructure?

This project has not directly contributed to improvements in local infrastructure.

15. What safeguarding procedures were used to protect local study participants and researchers?

All study processes, including data collection, storage, analysis, and sharing, followed the principles and guidelines laid down by the ethics committee of the Faculty of Medicine, Pharmacy and Dentistry, University of Bamako, Mali, and the ethics committee of the London School of Hygiene and Tropical Medicine, UK.

Figure S1: Theory of Change

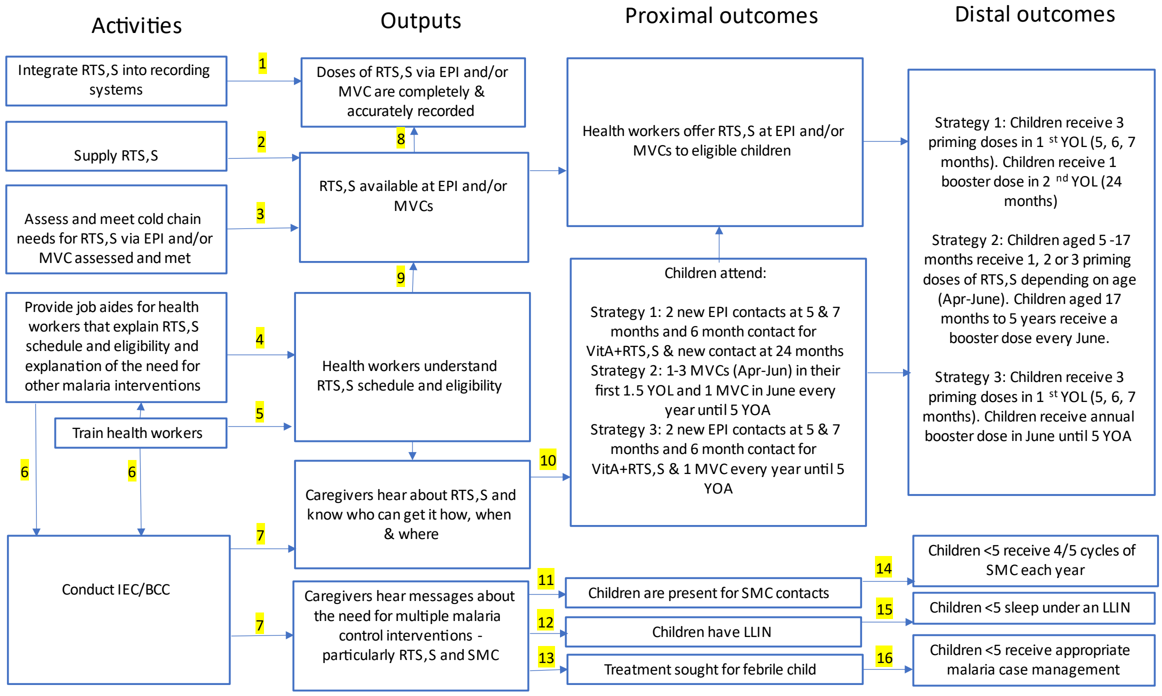

Table S1: Theory of change assumptions

Arrow	Assumption
1	- RTS,S can be integrated into the current recording systems at all levels (new systems are not required)<br>- Adequate numbers of new materials (registers, child vaccination cards) are printed and distributed to replace old materials in time for introduction<br>- HWs trained and understand how to record RTS,S
2	- Forecasting and logistics effective<br>- supply large quantities of RTS,S for around 3 months each year (strategy 2)<br>- supply RTS,S all year round in clinics with a large surge for annual MVC (strategy 3)
3	- Cold chain capacity at all levels that accommodates:<br>o RTS,S storage all year around (strategy 1)<br>o Large increase in storage needs for only part of the year (strategy 2)<br>o RTS,S storage all year with a surge for annual campaign (strategy 3)
4	- guidelines are clear and well-thought out, especially regarding eligibility<br>- job aides are clear and understandable<br>- sufficient number of job aides distributed<br>- Job aides are accessible to all HWs who need them<br>- HWs have to time to engage with the training and materials
5	- HWs understand training<br>- All necessary HWs are trained<br>- Additional refresher training is given<br>- Training is given at the appropriate time(s) during planning and implementation<br>- Supportive supervision is given
6	- HWs believe in the need for RTS,S (including all doses of RTS,S)<br>- HWs believe in need for RTS,S, SMC and LLINs<br>- HWs have time to conduct IEC/BCC
7	Effective messages and delivery channels are used, in terms of:

	- Access to messages<br>- Understandability of messages, in particular-<br>o Strategy 1: new vaccine contacts, including in 2nd YOL<br>o Strategy 2: Confusing eligibility of MVCs for priming doses<br>o Strategy 3: Receive different doses via different delivery systems<br>o What vaccine will protect against, need for multiple malaria interventions<br>- Impact of messages<br>- Timing of messages (particularly for MVCs and SMC)
8	- Caregivers retain and bring vaccination cards to every MVC/EPI contact<br>- There are registers with individual RTS,S receipt recorded if caregivers forget/lose cards<br>o Registers present at MVCs (strategies 2/3)<br>o Registers are accurate and up to date<br>- HWs at MVCs have time to check records (strategies 2/3)<br>- HWs able to accurately assess child age if no record
9	- HWs believe in RTS,S and need for all doses, and want to offer it at EPI contacts<br>- HWs decide to adhere to eligibility criteria (possibly bigger assumption in MVC setting)
10	- Acceptability of RTS,S<br>- Perceived need for RTS,S (alongside SMC and other malaria interventions)<br>- Perceived need for all doses of RTS,S<br>- Vaccination sites accessible (MVCs vs EPI clinics)<br>- Caregivers have time and resources needed to attend<br>- Good experiences of earlier doses so return to MVC/EPI for next dose<br>- Remember to attend EPI/MVC<br>o Large assumption for EPI 4th dose<br>MVCs more visible
11	- Caregiver wants child to receive both RTS,S and SMC<br>- Understands partial protection of both and additive effect<br>- Acceptability of SMC alone<br>- caregivers have time and resources to attend both RTS,S and SMC contacts each year<br>- Child is home at time of SMC delivery
12	- Caregiver understands need for multiple interventions and wants child to sleep under LLIN<br>- LLINs available and affordable/free
13	- Caregiver understands child can still get malaria despite RTS,S+SMC+LLINs
14	- Child eligible to receive SMC<br>- Day 2 and day 3 doses given to child by caregiver<br>- Adequate supplies of SPAQ
15	- Child prioritised for LLIN
16	- Access to HWs, diagnostics, treatment

HWs= health workers; RTS,S= RTS,S/AS01$_E$; IEC/BCC= information, education and communications/behaviour change communications; YOL= year of life; YOA= years of age; VitA= vitamin A

Figure S2: trial results figures used in interviews with EPI and NMCP programme managers and directors of health centres (a), and with other health workers, caregivers and community stakeholders (b)

a)

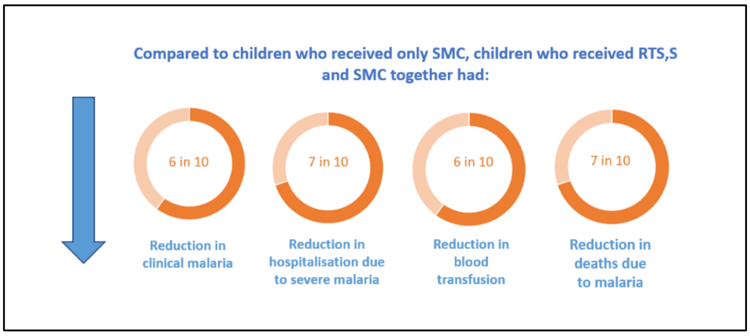

b)

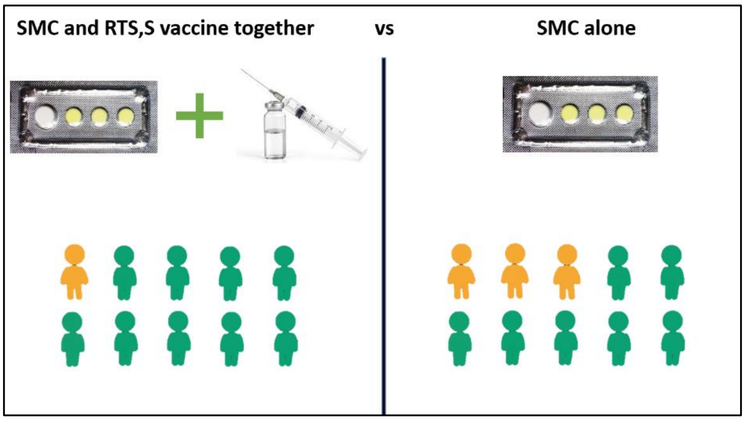

Table S2: The Standards for Reporting Qualitative Research (SRQR) checklist

**Title and abstract**	
1.Title	Includes a concise description of the topic of the study p1
2. Abstract	Abstract includes the background and purpose (aim), methods, results and conclusions p2
**Introduction**	
3. Problem formulation	Significance of the problem/phenomenon studied p3; relevant theory p4; problem statement p3
4. Purpose or research question	Purpose (aim) p3
**Methods**	
5. Qualitative approach and research paradigm	Qualitative approach and guiding theory (realist) p4-5, analytical framework p6
6. Researcher characteristics and reflexivity	Researcher characteristics and institutional background p1 and p6
7. Context	Study setting and salient contextual factors p4
8. Sampling strategy	Selection of study sites and stakeholders for in-depth interviews p5 and p8
9. Ethical issues pertaining to human subjects	Ethics approval from two ethics committees and informed consent p18
10. Data collection methods	Data collection procedures p5-6
11. Data collection instruments and technologies	Discussion guide details p4-5
12. Units of study	Type of participants and number of participants p5 and p8
13. Data processing	Data management prior to analysis p6
14. Data analysis	Description of coding process p6, verification by wider team and end of study workshop p4 and p6
15. Techniques to enhance trustworthiness	Analysis conducted and reviewed by multiple authors and participants of workshop to help validate the findings and interpretation p4 and p6
**Results/Findings**	
16. Synthesis and interpretation	Main findings p6-14
17. Links to empirical data	As this is a high level paper, presenting a synthesised summary of the qualitative findings amongst other results, no quotes from the interviews have been presented.
**Discussion**	
18. Integration with prior work, implications, transferability, and contributions to the field	Summary of main findings and how they connect to and elaborate on earlier scholarship p14-16. Discussion of generalisability p16. identification of unique contribution to scholarship p2-3 and p17.
19. Limitations	Limitations p16-17
**Other**	
20. Conflicts of interest	None to declare p17
21. Funding	PATH and UK Joint Global Health Trials p17
