# Supplementary material for: Delivery strategies for malaria vaccination in areas with seasonal malaria transmission
Source: BMJ Glob Health. 2023 May 5;8(5):e011838. doi: 10.1136/bmjgh-2023-011838 (PMC10163455; doi:10.1136/bmjgh-2023-011838)
Supplement: Supplementary data [file bmjgh-2023-011838supp002.pdf]

**Disclaimer : the English to French translation is the responsibility of the authors and BMJ can accept no liability for its accuracy / la traduction de l'anglais au français est responsabilité des auteurs, de conséquence BMJ ne va pas accepter aucune responsabilité lie'(ou par rapport) à son exactitude**

# Stratégies de distribution de la vaccination antipaludique dans les zones de transmission saisonnière du paludisme

**Liste d'auteurs :** *Jane Grant[1],*Halimatou Diawara[2], Seydou Traoré[2], Fatoumata Koita[2], Jessica Myers[1], Issaka Sagara[2], Daniel Chandramohan[1], Alassane Dicko[2], Brian Greenwood[1], Jayne Webster[1]
** Co-premiers auteurs*

1 Faculté des maladies infectieuses et tropicales, London School of Hygiene and Tropical Medicine, Londres, Royaume-Uni

2 Centre de recherche et de formation sur le paludisme (MRTC), Université des Sciences, des Techniques et des Technologies de Bamako , Mali

**Auteur correspondant :** Jane Grant, Faculté des maladies infectieuses et tropicales, London School of Hygiene and Tropical Medicine, Keppel St., London WC1E 7HT, Royaume-Uni (jane.grant2@lshtm.ac.uk).



Grant J, *et al. BMJ Global Health* 2023; 8:e011838. doi: 10.1136/bmjgh-2023-011838

**Mots clés :** vaccin contre le paludisme, RTS,S, paludisme saisonnier, stratégie de distribution, mise en œuvre, Mali

**RÉSUMÉ**

**Contexte :** La vaccination saisonnière avec le vaccin antipaludique RTS,S/AS01$_E$ administré en même temps que la chimioprévention du paludisme saisonnier (CPS) réduit considérablement le paludisme chez les jeunes enfants. L'OMS a recommandé l'utilisation du RTS,S/AS01$_E$, y compris la vaccination saisonnière, dans les zones de transmission saisonnière du paludisme. Cette étude visait à identifier les stratégies potentielles pour administrer RTS,S/AS01$_E$, et à évaluer les considérations et les recommandations pour l'administration de la vaccination contre le paludisme saisonnier au Mali, un pays où le paludisme est hautement saisonnier.

**Méthodes :** Des stratégies potentielles de distribution de RTS,S/AS01$_E$ dans les zones de paludisme saisonnier ont été identifiées grâce à une série de discussions de haut niveau avec les chercheurs de l'essai RTS,S/ AS01$_E$ plus CPS, des experts nationaux et internationaux en matière de vaccination et de paludisme, et grâce à l'élaboration d'une théorie du changement. Ces stratégies ont été explorées lors d'entretiens qualitatifs approfondis avec 108 participants, dont des responsables de programmes de paludisme et de vaccination au niveau national, régional et du district, des agents de santé, des gardiens/tuteurs d'enfants de moins de cinq ans et des acteurs communautaires. Un atelier national a été organisé pour confirmer les résultats qualitatifs et trouver un consensus sur une stratégie appropriée.

**Résultats :** Quatre stratégies de distribution ont été identifiées : la vaccination en fonction de l'âge via le Programme Elargi de Vaccination (PEV) ; la vaccination saisonnière via les campagnes de vaccination de masse du PEV ; une combinaison de doses de primovaccination en fonction de l'âge délivrées par les cliniques du PEV et de doses de rappel saisonnières délivrées par les campagnes de vaccination de masse ; et une combinaison de doses de primovaccination en fonction de l'âge et de doses de rappel saisonnières, toutes délivrées par les cliniques du PEV, qui était la stratégie préférée pour la distribution de RTS,S/AS01$_E$ au Mali identifiée lors de l'atelier national. Les participants ont recommandé que des interventions de soutien, notamment en matière de communication et de mobilisation, soient nécessaires pour que cette stratégie atteigne la couverture requise.

**Conclusions :** Quatre stratégies de distribution ont été identifiées pour l'administration de RTS,S/AS01$_E$ en même temps que la CPS dans les pays où la transmission du paludisme est saisonnière. Les composantes de ces stratégies de distribution ont été définies comme le calendrier de vaccination, le(s) système(s) de distribution et les interventions de soutien nécessaires pour que les stratégies soient efficaces. Il est nécessaire de poursuivre la recherche et l'évaluation de la mise en œuvre afin d'explorer comment, où, quand et quelle couverture effective peut être atteinte par ces nouvelles stratégies et leurs interventions de soutien.

## MESSAGES CLÉS

### Que sait-on déjà sur ce sujet ?

- La vaccination saisonnière avec le vaccin antipaludique RTS,S/AS01$_E$, administrée parallèlement à la chimioprévention du paludisme saisonnier, réduit considérablement le paludisme chez l'enfant et a été recommandée par l'OMS dans les zones de transmission saisonnière du paludisme.
- Le vaccin RTS,S/AS01$_E$ n'a été mis en œuvre que dans les zones non saisonnières par le biais d'une stratégie basée sur l'âge, dans le cadre du Programme Elargi de Vaccination (PEV), et seulement jusqu'à l'âge de deux ans.
- De nouvelles approches pourraient être nécessaires pour l'administration du RTS,S/AS01$_E$ dans les zones de transmission saisonnière du paludisme. Aucun autre vaccin infantile de routine n'est actuellement administré selon un calendrier saisonnier ou au-delà de l'âge de deux ans dans ces pays.

### Qu'apporte cette étude ?

- Cette étude élargit la réflexion actuelle en identifiant quatre stratégies possibles pour l'administration du RTS,S/AS01$_E$ dans les zones de transmission saisonnière du paludisme, définit les composantes d'une stratégie d'administration et prend en compte les stratégies de vaccination saisonnière et par âge ainsi que leurs systèmes de distribution.
- Cette étude présente les considérations nationales et le raisonnement qui a permis de déterminer la stratégie d'administration préférée, qui au Mali était des doses de primovaccination basées sur l'âge et des doses de rappel saisonnières annuelles, toutes administrées via le PEV de routine.
- Des interventions de soutien ont été identifiées qui seront nécessaires pour augmenter l'efficacité des stratégies au Mali.

### Comment cette étude pourrait-elle affecter la recherche, la pratique ou la politique ?

- Les décideurs et les responsables de la mise en œuvre peuvent utiliser les stratégies de distribution proposées et les résultats présentés dans cette étude, ainsi que d'autres recherches et considérations pratiques, économiques et contextuelles, pour prendre des décisions sur la distribution de RTS,S/AS01$_E$ dans les zones de transmission saisonnière du paludisme.
- Des recherches sur la mise en œuvre et l'évaluation des programmes sont nécessaires pour ces nouvelles stratégies de distribution et leurs interventions de soutien dans des contextes clairement définis afin de maintenir l'impact impressionnant obtenu dans les conditions de l'essai.

## CONTEXTE

La chimioprévention du paludisme saisonnier (CPS), c'est-à-dire l'administration mensuelle d'antipaludiques aux enfants de moins de cinq ans pendant la saison de transmission du paludisme, est un moyen efficace de prévenir le paludisme chez les jeunes enfants dans les régions où le paludisme est saisonnier, et elle est désormais largement déployée (1,2). Néanmoins, le paludisme reste la cause la plus fréquente de décès et d'hospitalisations chez les enfants de moins de cinq ans

dans de nombreuses zones saisonnières (3), et de nouvelles approches de la lutte contre le paludisme sont nécessaires. En 2021, l'OMS a recommandé l'utilisation généralisée du vaccin antipaludique RTS,S/AS01$_E$ dans les zones de transmission modérée à élevée du paludisme, précisant que les pays où la transmission du paludisme est saisonnière peuvent envisager de fournir le vaccin de façon saisonnière (4).

Dans le cadre du programme de mise en œuvre du vaccin antipaludique (MVIP), qui a introduit le RTS,S/AS01$_E$ dans trois pays à transmission pérenne en 2019, quatre doses du vaccin ont été intégrées au programme élargi de vaccination (PEV) de routine du pays selon un calendrier basé sur l'âge, en vaccinant les enfants jusqu'à 2 ans (5). Cependant, l'administration du vaccin contre le paludisme saisonnier aux enfants jusqu'à l'âge de cinq ans nécessite une nouvelle approche, car aucun autre vaccin infantile de routine n'est actuellement administré selon un calendrier saisonnier basé sur l'âge dans ces pays, et aucun vaccin infantile n'est administré systématiquement au-delà de l'âge de deux ans.

Cette étude visait à identifier les stratégies potentielles pour administrer le vaccin RTS,S/AS01$_E$ en même temps que la CPS dans les zones de transmission saisonnière du paludisme, à évaluer les perceptions des parties prenantes sur les stratégies et à développer des recommandations pour la mise en œuvre au Mali. L'étude fournit une première étape dans l'identification et le développement de stratégies de distribution pour le vaccin contre le paludisme saisonnier, et les considérations et recommandations clés pour sa distribution dans un pays avec le paludisme saisonnier.

## MÉTHODES

### Conception et composantes de l'étude

Cette étude comportait trois volets. Tout d'abord, les chercheurs ont identifié les stratégies potentielles d'administration du RTS,S/AS01$_E$ en même temps que la CPS dans les zones de paludisme saisonnier grâce à une série de discussions de haut niveau avec les chercheurs de l'essai RTS,S/ AS01$_E$ plus CPS, des experts nationaux et internationaux en matière de vaccination et de paludisme, et grâce à l'élaboration d'une théorie du changement. Ensuite, ces stratégies ont été explorées lors d'entretiens qualitatifs approfondis (IDI) avec les principales parties prenantes aux niveaux national, régional, du district, des établissements de santé et de la communauté. La collecte de données qualitatives comprenait des entretiens réalistes (6) afin d'explorer quelle stratégie de distribution fonctionne pour qui et dans quelles circonstances pour parvenir à une distribution efficace de RTS,S/AS01$_E$ en même temps que la CPS. Les approches réalistes sont fondées sur la théorie et reposent sur le principe que les interventions fonctionnent sur la base des décisions des individus, et que ces décisions sont motivées par des mécanismes déclenchés dans certains contextes et pas dans d'autres (7). Au fur et à mesure que l'étude et les discussions périphériques entourant la mise en œuvre de RTS,S/AS01$_E$ progressaient, ces stratégies ont été adaptées. Sur la base des données qualitatives et des discussions entre les chercheurs de l'étude et les experts mondiaux, une quatrième stratégie d'administration a été identifiée.

Enfin, après la collecte et l'analyse des données qualitatives, un atelier a été organisé à Bamako avec les principales parties prenantes du Programme national de lutte contre le paludisme (PNLP) et du PEV, ainsi que les principaux représentants de ces programmes dans les régions et districts de l'étude. Lors de l'atelier, les quatre stratégies de distribution et les résultats des données qualitatives ont été présentés, ainsi que les résultats d'efficacité et de sécurité des cinq années de l'essai

saisonnier RTS,S/AS01$_E$ plus CPS (8). Ces résultats ont été discutés afin de parvenir à un consensus sur la manière de distribuer le RTS,S/AS01$_E$ plus CPS au Mali.

**Site de l'étude**

L'étude a eu lieu au Mali. Pour la collecte des données au niveau des districts et des régions, deux districts et leurs régions respectives ont été inclus : les districts de Ouelessebougou et de Bougouni, qui se trouvent respectivement dans les régions de Koulikoro et de Sikasso. En plus de la collecte de données dans deux districts et leurs régions respectives, des entretiens approfondis et l'atelier ont été organisés avec des parties prenantes au niveau national au Mali. En outre, des discussions au niveau mondial ont contribué à l'identification des stratégies de mise en œuvre, comme décrit ci-dessous. Ouelessebougou et Bougouni sont des districts semi-ruraux avec des niveaux élevés d'analphabétisme, et l'agriculture comme principale occupation. L'essai du RTS,S/AS01$_E$ plus CPS a été mené dans certaines parties de ces districts de 2017 à 2021 (8). Le paludisme est très saisonnier, la plupart des cas se produisant de juillet à novembre. Dans les districts de l'étude, les régions et au niveau national, le paludisme est la première cause de consultations externes, d'hospitalisations et de décès chez les enfants de moins de cinq ans (9). Quatre cycles mensuels de CPS sont délivrés par le PNLP via des campagnes de porte-à-porte de juillet à octobre, avec certaines parties du pays pilotant actuellement l'ajout d'un cinquième cycle. Neuf vaccinations infantiles différentes sont administrées systématiquement par le programme PEV dans les centres de santé et par la stratégie avancée . Au Mali, les campagnes de vaccination de masse sont également utilisées en réponse aux épidémies, pour introduire de nouveaux vaccins, ou lorsque la couverture de routine est faible. La couverture du PEV est relativement élevée au Mali, avec une estimation de 77 % des enfants recevant le DTP-3 dans le pays (10).

**Identification des stratégies de livraison et développement de la théorie du changement**

Les stratégies potentielles d'administration du vaccin ont été identifiées au début de l'étude en septembre 2021 par une série de discussions de haut niveau avec les investigateurs de l'essai RTS,S/AS01$_E$ plus CPS, des experts internationaux et nationaux en matière de vaccination et de paludisme. Une théorie du changement (TdC) a été utilisée (en tant qu'aide à la conception de programmes) pour étudier l'adéquation des stratégies dans le cadre d'un programme national potentiel (11). Il s'agissait notamment d'examiner les activités qui seraient nécessaires pour générer les résultats requis par le biais desquels les résultats de la stratégie seraient atteints, et donc les avantages et les défis relatifs des stratégies spécifiques et de leurs composantes (figure S1 et tableau S1). La TdC a été élaboré sur la base de l'expérience des investigateurs de l'étude dans la mise en œuvre d'interventions similaires, complétée par une revue de la littérature sur les stratégies de mise en œuvre d'autres interventions, y compris les vaccins du PEV de routine, les campagnes de vaccination, d'autres campagnes verticales, y compris les campagnes sur la CPS et la nutrition, et l'étude pilote RTS,S/AS01E.

Les stratégies d'administration du vaccin sont composées de deux éléments principaux : le calendrier de vaccination et le système de distribution (figure 1). Le calendrier de vaccination comprend le nombre de doses de vaccin, les âges cibles et le fait que les vaccins soient administrés selon un calendrier basé sur l'âge ou un programme saisonnier (basé sur le calendrier). Le RTS,S/AS01$_E$ est administré sous forme de trois injections de primovaccination à un mois d'intervalle au cours de la première année, suivies de doses de rappel. Dans le cadre de l'essai MVIP, une dose de rappel a été administrée à l'âge de 24 mois, tandis que dans l'essai RTS,S/AS01$_E$ plus CPS, quatre doses de rappel saisonnières annuelles ont été administrées jusqu'à ce que les enfants atteignent l'âge de cinq ans,

âge auquel la CPS s'arrête. Le deuxième élément est le(s) système(s) de distribution utilisé(s) pour administrer les doses.

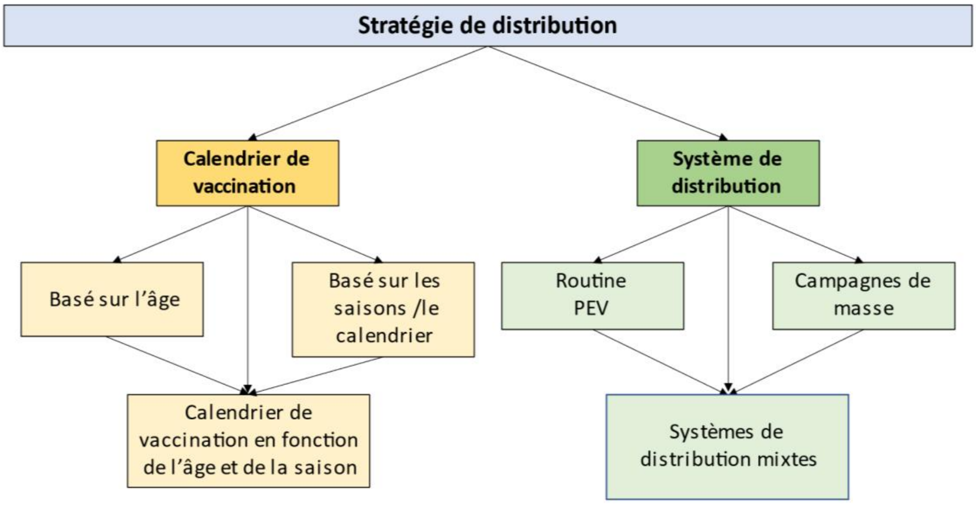


Figure 1 : Composantes des stratégies de distribution du vaccin RTS,S/AS01$_E$ dans les zones de paludisme saisonnier

**Collecte et analyse des données qualitatives**

Un échantillonnage à dessein a été utilisé pour sélectionner : les principaux responsables du PNLP et du PEV au niveau national, régional et du district ; différents cadres de agents de santé impliqués dans l'administration des vaccins du PEV et de la CPS ;; les parties prenantes concernées dans chaque communauté ; et les gardiens/tuteurs d'enfants de moins de cinq ans. Les agents de santé ont été échantillonnés dans huit centres de santé communautaires sélectionnés dans les districts de Bougouni et de Ouelessebougou. Ces formations sanitaires ont été sélectionnées de manière ciblée parmi 61 formations sanitaires dans les districts afin d'inclure des variations entre les formations sanitaires, y compris si elles se trouvaient ou non dans les sites d'essai RTS,S/AS01$_E$ plus CPS, si elles étaient situées dans un milieu urbain/rural, et les formations sanitaires avec des couvertures PEV relativement plus élevées ou plus faibles. Dans chaque établissement de santé, les agents de santé qui travaillaient sur les programmes PEV et CPS, y compris le directeur de l'établissement, ont été sélectionnés, l'objectif étant de sélectionner environ quatre agents de santé par établissement de santé. Les gardiens/tuteurs d'enfants ont été échantillonnés dans les zones de recrutement des huit établissements de santé sélectionnés, et ont été choisis de manière ciblée pour inclure la variation du sexe, de la distance par rapport à l'établissement de santé et de l'alphabétisation. Les gardiens/tuteurs d'enfants de l'essai et ceux qui ne l'étaient pas ont été inclus afin d'obtenir le point de vue de ceux qui avaient ou n'avaient pas reçu le vaccin RTS,S/AS01$_E$ (ou le vaccin témoin), et pour éviter tout biais lié à l'inclusion dans l'essai.

Des guides de discussion pour les IDI ont été élaborés sur la base des stratégies de distribution et de la théorie du changement. En outre, plusieurs cadres théoriques ont été utilisés pour formuler les questions permettant de sonder les perceptions des participants sur les différentes stratégies de distribution, notamment les éléments constitutifs des systèmes de santé de l'OMS (12), la théorie de la diffusion des innovations de Roger (13) et le cadre de faisabilité de Bowen (14).

Différents guides de discussion ont été utilisés pour les responsables de programmes de santé, les agents de santé, les parents/tuteurs d'enfants et les parties prenantes communautaires. Au début des entretiens, le contexte de l'essai sur la vaccination saisonnière du vaccin RTS,S/AS01$_E$ plus CPS a été décrit, et les résultats de l'essai ont été présentés à l'aide d'un graphique (Figure S2). Les stratégies d'administration ont ensuite été présentées par les enquêteurs aux personnes interrogées, soit à l'aide de chiffres, soit verbalement. Après cela, l'enquêteur a posé une série de questions écrites, ouvertes et d'approfondissement pour faciliter la discussion sur chaque stratégie. Enfin, le participant a été invité à comparer les stratégies et à donner sa préférence globale et sa justification pour la stratégie à utiliser pour administrer le RTS,S/AS01$_E$. Le niveau de détail dans lequel les résultats de l'essai et les stratégies d'administration ont été présentés et discutés variait selon le groupe de participants.

Guidés par les guides de discussion, les enquêteurs ont interrogé le contexte dans lequel le vaccin RTS,S/AS01$_E$ et la CPS seraient administrés, et comment cela affectait les perceptions et les recommandations des participants pour l'administration du vaccin. Cette interrogation a été soutenue chez les agents de santé, les gardiens/tuteurs d'enfants et les parties prenantes de la communauté par la présentation et la discussion/validation des configurations contexte-mécanisme-résultat (CMO) qui représentent les théories sur les facteurs (contextes) et les mécanismes qui conduisent aux recommandations pour l'exécution des interventions. Les CMO ont été élaborées à partir des TdC et de l'examen des données préliminaires issues des notes de terrain prises lors des IDI des responsables de programme.

Les entretiens ont été menés en français et en bambara par quatre chercheurs formés au Centre de recherche et de formation sur le paludisme (MRTC). Tous les entretiens ont été enregistrés numériquement. Pendant les IDI, un deuxième chercheur a pris des notes sur le terrain des principaux points et des observations clés de l'entretien. Ces notes ont été utilisées pour examiner les points clés émergents des données et améliorer le processus d'entretien. Les entretiens en français ont été transcrits *mot à mot* et les entretiens en bambara ont été simultanément transcrits et traduits en français. Toutes les transcriptions ont ensuite été traduites en anglais, et importées dans NVivo pour le codage et l'analyse. Les transcriptions ont été rendues anonymes, mais le numéro de l'entretien et le groupe de participants ont été conservés pour faciliter l'analyse. Les transcriptions ont été codées par deux des chercheurs de l'étude du LSHTM à l'aide d'une approche d'analyse de cadre avec un cadre de codage initial développé sur la base des thèmes clés des guides d'entretien (15). Ces thèmes ont ensuite été complétés inductivement par des sous-thèmes au fur et à mesure qu'ils étaient identifiés dans les données. Pendant l'analyse, des notes détaillées ont été enregistrées par les deux codeurs afin d'éclairer l'interprétation des résultats. Le codage et les résultats synthétisés ont été discutés entre les chercheurs du LSHTM et du MRTC à plusieurs reprises au cours de l'analyse afin de vérifier le codage et d'assurer la crédibilité et la confirmabilité des résultats.

Les Normes de présentation des recherches qualitatives ont été utilisés pour garantir une présentation rigoureuse des résultats qualitatifs (tableau S2).

**Atelier national des parties prenantes**
Un atelier a été organisé à Bamako en juillet 2022 avec les principales parties prenantes du PNLP et du PEV, et les principaux représentants de ces programmes dans les régions et districts de l'essai. Lors de l'atelier, les quatre stratégies d'administration et les thèmes des données qualitatives ont été présentés, ainsi que les résultats d'efficacité et de sécurité à cinq ans de l'essai sur la vaccination saisonnière du vaccin RTS,S/AS01$_E$ plus CPS (8). Les résultats de l'étude qualitative, y compris les trois stratégies d'administration initiales, ainsi que la quatrième stratégie d'administration nouvellement

identifiée, ont été utilisés pour discuter et parvenir à un consensus entre les parties prenantes sur la manière d'administrer le RTS,S/AS01$_E$ plus CPS au Mali. En outre, à travers la présentation et la discussion des résultats qualitatifs, l'atelier a contribué à valider les résultats et l'interprétation des données. L'atelier a fait l'objet d'un procès-verbal.

**Participation des patients et du public**

Les points de vue et les expériences des gardiens/tuteurs d'enfants, des acteurs communautaires et des agents de santé ont été recherchés en tant que participants à cette étude, et ces groupes n'ont pas été impliqués dans la conception, la conduite, le rapport ou les plans de diffusion de cette étude. Les responsables des programmes de lutte contre le paludisme et de vaccination au Mali ont contribué à la question de recherche, à la conception de l'étude et à sa diffusion. Une déclaration de réflexivité de l'auteur est fournie dans l'annexe supplémentaire.

## RÉSULTATS

### Stratégies de mise en œuvre pour RTS,S/AS01$_E$ aux côtés de CPS

Les trois premières stratégies de distribution (1 à 3) identifiées au début de l'étude, et discutées lors des IDI, étaient le PEV de routine basé sur l'âge (stratégie 1), les campagnes saisonnières de vaccination de masse (CVM) (stratégie 2) et les systèmes de distribution mixtes basés sur l'âge et la saison (stratégie 3) (Figure 2). D'après les perceptions des trois stratégies discutées au cours des IDIs et les discussions entre les investigateurs de l'essai et les experts mondiaux, il est apparu que la mise en œuvre des CVM posait d'importants problèmes de faisabilité, mais que la mise en œuvre de doses de rappel saisonnières suscitait un vif intérêt. C'est pourquoi une nouvelle stratégie, le PEV de routine basé sur l'âge et la saison (stratégie 4), a été élaborée (Figure 2d).

Dans les quatre stratégies, la CPS est administrée comme d'habitude par le PNLP via quatre campagnes mensuelles pendant la saison de transmission du paludisme. Dans chacune des stratégies, les enfants peuvent recevoir la première dose de RTS,S/AS01$_E$ à partir de l'âge de cinq mois, et avec un minimum de quatre semaines entre les doses. Dans les stratégies 2 à 4, il y a un intervalle de 12 mois entre les doses de rappel.

***a) Stratégie 1 :** PEV **de routine basé sur l'âge***

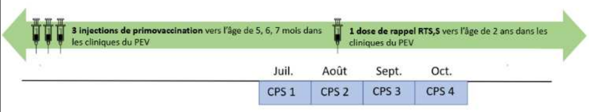


**Programme de vaccination :** trois doses de primovaccination et une dose de rappel. Toutes les doses sont administrées selon un calendrier basé sur l'âge, les doses de primovaccination étant administrées tous les mois à partir de l'âge de 5 mois, et la dose de rappel vers l'âge de 18 à 24 mois.

**Système de distribution :** Toutes les doses administrées dans les cliniques de vaccination du PEV de routine toute l'année.

| | |
|---|---|
| ***b) Stratégie 2 : CVM saisonnières*** 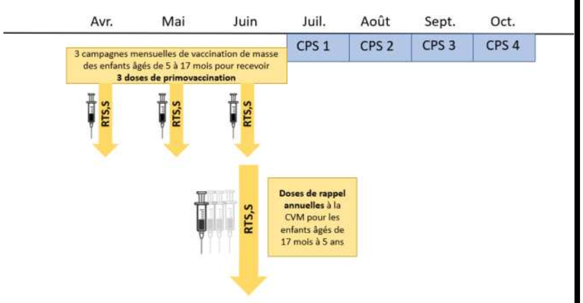  | **Programme de vaccination :** trois doses de primovaccination et quatre doses de rappel annuelles. Toutes les doses sont administrées de manière saisonnière/conformément à un calendrier. Les enfants doivent avoir entre 5 et 17 mois pour recevoir les doses de primovaccination, et peuvent recevoir les rappels annuels jusqu'à l'âge de cinq ans.<br><br>**Système de distribution :** toutes les doses sont administrées via les CVM. Les doses de primovaccination sont administrées lors de trois CVM mensuelles consécutives juste avant la saison de transmission du paludisme (par exemple, avril-juin). Les doses de rappel sont administrées lors du troisième CVM, juste avant la saison de transmission. |
| ***c) Stratégie 3 : Systèmes de distribution basés sur l'âge et mixtes saisonniers*** 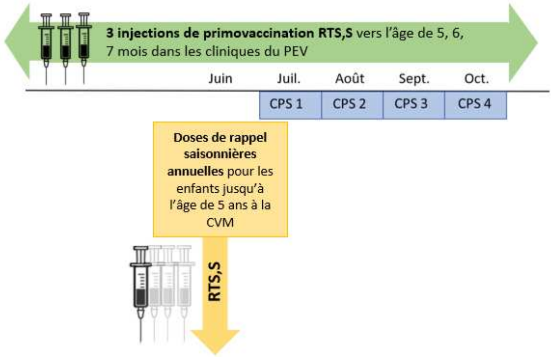  | **Calendrier de vaccination** : combinaison de la vaccination en fonction de l'âge et de la vaccination saisonnière, avec trois doses de primovaccination et quatre doses de rappel annuelles. Les doses de primovaccination sont administrées selon un calendrier basé sur l'âge, et les doses de rappel sont administrées de façon saisonnière. Les enfants peuvent recevoir les doses de primovaccination tous les mois à partir de l'âge de 5 mois, comme dans la stratégie 1, et peuvent recevoir des rappels annuels jusqu'à l'âge de cinq ans, comme dans la stratégie 2.<br><br>**Système de distribution :** Les doses de primovaccination sont administrées dans les cliniques du PEV de routine toute l'année (comme dans la stratégie 1). Les doses de rappel sont administrées lors d'une CVM annuelle, juste avant la saison de transmission (comme dans la stratégie 2). |
| ***d) Stratégie 4 : PEV de routine basé sur l'âge et la saison*** 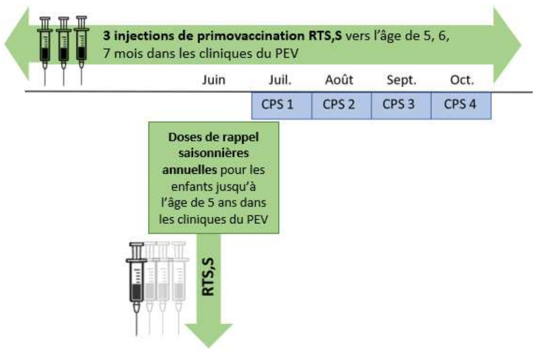  | **Programme de vaccination :** identique à la stratégie 3<br><br>**Système de distribution :** Toutes les doses sont administrées dans les cliniques du PEV de routine. Les doses de primovaccination sont administrées toute l'année. Les soignants doivent amener leurs enfants à la clinique du PEV pour les rappels annuels dans le mois précédant la saison de transmission du paludisme (par exemple, en juin). |

**Clé :**

Doses de RTS,S délivrées par les cliniques PEV de routine

Doses de RTS,S délivrées par des campagnes de vaccination de masse

Cycle de CPS assuré par le PNLP

Figure 2 : Stratégies potentielles pour la mise en œuvre de RTS,S/AS01$_E$ aux côtés de la CPS

**Perceptions des stratégies de mise en œuvre et recommandations pour la mise en œuvre**

Cent huit participants ont été interrogés (Tableau 1). Les résultats sont présentés en fonction des trois principaux groupes de participants : les responsables de programmes, les agents de santé, et les participants au niveau de la communauté (gardiens/tuteurs d'enfants et les acteurs communautaires (Figure 3). Les perceptions des avantages et des difficultés des stratégies, ainsi que

les recommandations relatives à leur mise en œuvre, étaient similaires chez les participants de l'essai RTS,S/AS01$_E$ + CPS et chez les participants des sites qui n'ont pas fait l'objet de l'essai.

Tableau 1 : Participants aux entretiens approfondis

| Type de participant | Total |
|---|---|
| Responsables des programmes PEV et paludisme | |
| Niveau national | 8 |
| Niveau régional | 7 |
| Niveau de district | 10 |
| Agents de santé | 32 |
| Les soignants des enfants inscrits à l'essai RTS,S/AS01$_E$ + CPS | 17 |
| Les soignants d'enfants de moins de cinq ans qui ne participent pas à l'essai | 26 |
| Parties prenantes communautaires* | 8 |
| Total | 108 |

* les chefs, les membres des associations locales de santé, les responsables des groupes de femmes, les responsables des groupes de jeunes et les responsables des associations d'agriculteurs.

Figure 3 : Avantages et défis des trois stratégies de distribution proposées (1-3), selon les groupes de participants

***Stratégie 1 : PEV de routine basé sur l'âge***

L'un des principaux avantages de cette stratégie décrite par tous les groupes est qu'elle intègre pleinement l'administration de RTS,S/AS01$_E$ dans un programme de routine existant, en tirant parti de l'infrastructure et des ressources du PEV, ce qui rend la stratégie moins coûteuse et plus durable. Les participants ont également déclaré que les agents de santé et les communautés connaissent déjà le PEV et y sont habitués, et que de nombreux enfants sont atteints par ce moyen. De plus, les responsables de programme et les agents de santé ont discuté de la manière dont ils ont précédemment introduit avec succès de nouveaux vaccins dans le programme. Les soignants et les acteurs communautaires ont estimé que cette stratégie serait relativement facile à mettre en œuvre, étant donné que le PEV est déjà une habitude pour la plupart des soignants, qu'il est bien accepté et qu'on lui fait confiance, les vaccins du PEV étant appréciés et considérés comme efficaces ayant réussi à réduire l'ampleur des maladies comme la rougeole. Ils pensent que si le RTS,S/AS01$_E$ est administré par le biais de ce programme de confiance, cela donnera de la crédibilité au nouveau vaccin et réduira les rumeurs négatives. Les responsables de programme et les agents de santé espéraient également que l'introduction du vaccin dans le PEV aurait un effet bénéfique sur l'ensemble du programme en raison du fardeau du paludisme dans ces communautés et de la demande de vaccin contre le paludisme, et que les nouveaux contacts RTS,S/AS01$_E$ offriraient de nouvelles opportunités de rattrapage des autres vaccins manquants du PEV, augmentant ainsi la couverture du PEV.

Tous les groupes ont également discuté des avantages de l'administration des trois doses de primovaccination seules lors des nouveaux contacts de vaccination. Les responsables du programme et les agents de santé ont apprécié le fait que cela rendrait le programme PEV plus continu et plus facile à retenir pour les soignants, les enfants venant presque tous les mois dès leur première année de vie. Les agents de santé ont ajouté qu'il y a beaucoup de vaccins du PEV dans les trois premiers mois de la vie, et qu'il est bénéfique d'ajouter RTS,S après une courte pause de cette période chargée. De nombreux soignants ont préféré recevoir le RTS,S/AS01$_E$ seul lors des nouveaux

contacts, car ils craignaient que les effets secondaires s'aggravent lorsque plusieurs vaccins sont administrés ensemble. De plus, s'il est administré lors des nouveaux contacts, davantage de personnes sauront que leur enfant reçoit le vaccin contre le paludisme, plutôt que plusieurs « vaccins du PEV ». Les agents de santé préféraient que les nouveaux vaccins soient administrés seuls afin de pouvoir surveiller plus facilement les effets secondaires.

Les participants ont souligné les difficultés à ajouter les nouveaux contacts du PEV, en particulier les doses de rappel. Pour les doses de primovaccination, les responsables du programme et les agents de santé ont déclaré qu'il serait difficile d'inciter les soignants à amener leurs enfants à la clinique pour les nouveaux contacts, car il y a déjà une perte de suivi importante dans le programme et beaucoup d'enfants ne viennent pas entre 3 et 9 mois, lorsqu'ils reviennent pour la vaccination contre la rougeole (MCV). Certains soignants et acteurs communautaires craignaient que les autres pensent que cela représente trop de contacts et de vaccins, étant donné les obstacles existants à la participation au PEV. Les participants ont également déclaré qu'il serait très difficile de faire revenir les enfants plus âgés pour la quatrième dose après le long intervalle qui sépare la troisième dose et que de nombreux soignants considèrent que le PEV se termine à l'âge de 9 mois. À ce moment-là, de nombreux soignants peuvent ne pas se souvenir de la nécessité de retourner à la clinique et peuvent avoir perdu leur carte de vaccination. Les participants ont suggéré que des activités supplémentaires seraient nécessaires pour établir les nouveaux contacts, en particulier pour la dose de rappel, y compris une sensibilisation et une mobilisation intensives avec une forte implication des bénévoles de la santé communautaire, des rappels systématiques, la recherche des personnes défaillantes, et éventuellement la fourniture d'autres motivations, comme des moustiquaires. Certains participants ont suggéré que, si possible, les doses de primovaccination soient administrées aux contacts existants et que l'âge de la dose de rappel soit abaissé, et qu'elles soient administrées en combinaison avec le MCV-2 à 15 mois. Les agents de santé ont également exprimé des inquiétudes quant à l'augmentation de leur charge de travail si cette stratégie est adoptée, y compris la réalisation d'activités supplémentaires nécessaires pour s'assurer que les soignants assistent aux contacts.

***Stratégie 2 : CVM saisonnières***

Un avantage majeur discuté par les participants était la capacité des CVM à atteindre une couverture élevée très rapidement ; les sites de vaccination plus accessibles et plus proches des communautés, ainsi que les mobilisations et communications intensives qui accompagnent les campagnes, étaient censés atteindre de nombreux enfants, y compris ceux qui ne participeraient pas au PEV. Les soignants et les acteurs communautaires ont expliqué que les communications intensives sont motivantes, ce qui entraîne une forte sensibilisation. Les soignants se sentiraient motivés et gagneraient en confiance en voyant d'autres personnes emmener leurs enfants à la campagne pour un nouveau vaccin. La couverture élevée, ainsi que la forte protection attendue des quatre doses de rappel saisonnières, se traduiraient par un effet protecteur visible du vaccin, maintenant une couverture élevée des doses ultérieures.

Malgré ces avantages, cette stratégie a suscité de grandes inquiétudes, notamment en ce qui concerne les ressources nécessaires et l'impact de l'organisation de trois campagnes annuelles de CVM, immédiatement avant quatre ou cinq campagnes mensuelles de CPS. Les responsables du programme ont considéré que les exigences importantes en matière de ressources financières et humaines pour les CVM constituaient un défi majeur, et ont rapporté que certains districts avaient déjà du mal à mobiliser des ressources pour organiser quatre campagnes mensuelles de CPS. En outre, les responsables de programmes nationaux ont évoqué la manière dont les campagnes devraient être financées par les partenaires et le fait qu'ils préféraient ne pas être financièrement

dépendants de partenaires qui ne les soutiendraient pas indéfiniment. En outre, lorsque plusieurs partenaires soutiennent un programme dans différents domaines, comme c'est le cas pour la CPS, le programme manque de cohésion. En outre, les responsables du programme et les agents de santé ont exprimé des inquiétudes quant à la charge que ces sept ou huit mois intensifs de campagnes contre le paludisme feraient peser sur la charge de travail des agents de santé et des parents et sur le fonctionnement du système de santé au sens large. Les agents de santé ont noté que les centres de santé et les cliniques du PEV sont souvent vides pendant les jours de campagne. Cependant, les agents de santé ont suggéré que les impacts négatifs des campagnes pourraient être minimisés s'ils étaient bien organisés et si des agents de santé qualifiés supplémentaires étaient engagés. En outre, la plate-forme de la campagne pourrait être utilisée pour fournir d'autres interventions, comme le MCV-2.

Si les participants ont apprécié la valeur de l'administration du vaccin saisonnier, tous les groupes ont exprimé des inquiétudes quant à la possibilité pour les enfants de ne recevoir le vaccin qu'à un seul moment de l'année. Les participants n'ont pas apprécié que certains enfants doivent attendre d'avoir l'âge requis au moment de la campagne pour recevoir leur première dose, et que ceux qui n'avaient pas encore l'âge requis pour recevoir les doses de primovaccination, ou qui ont manqué la campagne, ne soient pas protégés pendant la saison de transmission, d'autant plus que le mois de juin est une période chargée pour le travail sur le terrain.  Presque tous les responsables de programme et les agents de santé pensaient qu'une vaccination de rattrapage serait nécessaire, soit par le biais du programme PEV de routine et du dépistage avec orientation des enfants qui n'ont pas assisté à la campagne, soit par une distribution sur site fixe en même temps que la CPS, ce qui entraînerait des coûts supplémentaires et des difficultés opérationnelles.

Les responsables du programme et les agents de santé ont estimé qu'il serait difficile de déterminer l'âge auquel les enfants sont éligibles pour les doses de primovaccination, au cours de la CVM, et si les enfants ont reçu les doses de primovaccination nécessaires pour pouvoir recevoir une dose de rappel. Cela a été considéré comme un obstacle majeur car de nombreux soignants perdent ou n'apportent pas leurs cartes de vaccination ; les agents de santé sont très occupés pendant les CVM et n'ont pas le temps de vérifier les registres ; ils sont poussés par les soignants à vacciner les enfants inéligibles. Les responsables des programmes nationaux ont déclaré que cela était particulièrement difficile dans les zones où se trouvent des personnes déplacées à l'intérieur du pays, car elles n'ont souvent pas leur carte de vaccination et ne sont pas inscrites sur les registres.

***Stratégie 3 : Systèmes de distribution basés sur l'âge et mixtes saisonniers***

Cette stratégie a été considérée comme combinant les principaux avantages des deux stratégies décrites ci-dessus, en termes de calendrier de vaccination et de systèmes de distribution. Dans cette stratégie, les nourrissons reçoivent les doses de primovaccination au début de leur vie par le biais du PEV lorsqu'ils atteignent l'âge d'éligibilité. Les soignants apprécient de recevoir les doses de primovaccination via le PEV lorsque leurs enfants sont jeunes et qu'ils ont l'habitude de participer au PEV. Ensuite, les enfants reçoivent des rappels saisonniers efficaces, potentiellement jusqu'à l'âge de cinq ans, par l'intermédiaire des CVM, ce qui permet d'atteindre une couverture élevée pour ces doses. De nombreux soignants ont apprécié l'idée que les doses ultérieures soient administrées plus près de chez eux, par le biais de CVM bien connues, car il serait difficile d'emmener les enfants plus âgés à la clinique du PEV, car à ce stade, ils pourraient oublier que c'est nécessaire. En outre, de nombreuses mères ont des enfants plus jeunes à ce stade, et se concentrent davantage sur leur santé. Certains soignants ont également mentionné qu'il serait physiquement difficile d'emmener plusieurs enfants à la clinique du PEV.

Les autres avantages de cette stratégie étaient que les campagnes de rappel pouvaient être utilisées pour rattraper les enfants qui avaient manqué une partie ou la totalité des doses de primovaccination du PEV de routine mais qui étaient encore dans l'âge autorisé, augmentant ainsi la couverture de ces doses. De plus, pour les enfants qui manquent la campagne de rappel, il serait possible de fournir une vaccination de rattrapage, puisque le vaccin se trouve déjà dans les cliniques du PEV de routine pour être référé. Les responsables du programme ont suggéré que ces enfants puissent être identifiés en utilisant les registres avec l'aide des volontaires de santé communautaire, ou en vérifiant les cartes de vaccination lors de la distribution de CPS.

Le principal défi discuté pour cette stratégie était le besoin de ressources supplémentaires et la charge pour le système de santé d'ajouter ne serait-ce qu'une seule CVM annuelle, ce qui entraînerait 5 à 6 mois consécutifs de campagnes de lutte contre le paludisme chaque année. D'autres défis perçus par les agents de santé étaient que certains soignants seraient déroutés par le fait que le même vaccin soit administré à la fois par le PEV de routine et par la CVM et par la nécessité de se rendre aux deux pour des doses différentes.

***Recommandations sur les stratégies 1-3 des IDIs***

Alors que des défis et des avantages similaires ont été discutés par toutes les catégories de participants, les recommandations finales quant à la stratégie à utiliser pour délivrer le RTS,S/$AS01_E$ en même temps que la CPS, et la justification de cette décision, variaient entre les groupes de participants, y compris entre les différents niveaux de responsables de programmes (Tableau 2).

La majorité des responsables de programmes nationaux ont recommandé des systèmes de distribution mixtes en fonction de l'âge et de la saison (stratégie 3), car l'utilisation du PEV de routine pour les doses de primovaccination est durable et améliorerait la couverture du PEV, et une forte protection serait assurée par les doses de rappel saisonnières qui bénéficieraient d'une couverture élevée de la part des CVM. En outre, le rattrapage au niveau des CVM saisonniers augmenterait la couverture des doses de primovaccination. Les responsables du programme national ont estimé que la stratégie du PEV de routine (1) serait plus durable et moins chère, mais ils ne l'ont pas recommandée car elle offrirait une protection moindre du fait qu'elle ne serait pas saisonnière et qu'elle ne couvrirait que l'âge de deux ans. Malgré cela, la majorité des responsables de programmes régionaux et de district ont recommandé cette stratégie car elle s'intègre mieux au système actuel, et ils se sont inquiétés des pressions qu'une CVM annuelle (stratégie 3) ferait peser sur les établissements de santé communautaires. Les responsables de programme à tous les niveaux ont déclaré que la stratégie de la CVM (2) n'était pas réalisable en raison des coûts très élevés et de la charge sur le système de santé.

Les recommandations des agents de santé étaient plus divisées entre les stratégies. Cependant, la majorité recommandait le PEV de routine basé sur l'âge (1) en raison de la facilité et du faible coût d'utilisation du programme existant. La deuxième recommandation la plus fréquente était celle des systèmes de distribution mixtes par âge et par saison (3) pour des raisons similaires à celles des responsables de programme. Quelques agents de santé ont recommandé les CVM saisonnières (2) avec leur forte composante de communication et leur facilité d'accès pour les soignants plus ruraux, mais la majorité a trouvé cette stratégie moins acceptable en raison des coûts et de la charge de travail importants, et de la perturbation des activités de routine.

Certains responsables de programme et agents de santé ont également suggéré que les RTS,S/$AS01_E$ soient d'abord dispensés par les CVM, puis totalement ou partiellement intégrés au PEV de routine. Cela suit un modèle commun d'introduction de vaccins au Mali et les fortes mobilisations et

communications qui seraient nécessaires pour que les CVM réussissent aideraient à sensibiliser et à faire accepter le nouveau vaccin, et à développer la couverture. Comme les CVM ne seraient employés que pendant un ou deux ans, la question de la durabilité ne se poserait pas, et certains participants ont préféré utiliser les campagnes de cette manière pour soutenir le programme de routine, plutôt que de les mettre en place comme des programmes parallèles.

La majorité des soignants et des parties prenantes de la communauté ont préféré la stratégie des systèmes de distribution mixtes basés sur l'âge et la saison (3) car les soignants apprécient la combinaison de la protection précoce et saisonnière, et sont habitués au PEV pour les jeunes enfants, les CVM étant un moyen plus facile d'accéder aux doses ultérieures. Certains participants ont recommandé la stratégie du PEV de routine (1) en raison de la facilité et de leur confiance dans le programme de routine. Seuls deux soignants ont recommandé la stratégie de CVM (2), mais ce groupe de participants n'a pas trouvé cette stratégie inacceptable, contrairement aux responsables du programme et aux agents de santé. Les soignants et les parties prenantes ont fréquemment déclaré qu'en dépit de leurs préférences, ils trouveraient l'une ou l'autre de ces stratégies acceptable en raison du fardeau du paludisme et de l'importance du vaccin antipaludique pour eux, mais ils ont souligné la nécessité d'une bonne communication et d'une bonne compréhension pour que ces stratégies fonctionnent.

**Recommandations sur les stratégies 1-4 de l'atelier national**

***Stratégie 4 : PEV de routine basé sur l'âge et la saison***

Au total, 15 acteurs ont participé à l'atelier national qui s'est tenu à Bamako le 29 juillet 2022, dont 6 représentants du PNLP et du PEV national, 4 du PNLP et du PEV de chacune des régions et districts étudiés et un chercheur en santé publique. Après avoir examiné les résultats de l'efficacité de l'essai sur cinq ans, les participants ont estimé que les quatre doses en fonction de l'âge (stratégie 1) ne cadraient pas bien avec les preuves montrant l'efficacité d'une stratégie saisonnière à sept doses, vaccinant les enfants jusqu'à cinq ans.

Dans l'ensemble, les participants à l'atelier ont recommandé d'utiliser le PEV de routine basé sur l'âge et la saison (stratégie 4) pour mettre en œuvre le vaccin. Cette décision a été prise en raison des problèmes de faisabilité que posent les CVM, et du désir d'utiliser les systèmes existants pour la distribution, afin de réduire les coûts et d'améliorer la durabilité. Ce point a été souligné par les participants au niveau des districts, qui ont déclaré qu'il y avait trop de campagnes dans leurs districts et qu'ils craignaient que les établissements de santé communautaires ne soient pas en mesure de faire face à une autre campagne. Cependant, les participants ont partagé leurs inquiétudes sur le fait que la faible mobilisation dans cette stratégie entraînerait une faible couverture, car les soignants devraient amener les enfants jusqu'à cinq ans chaque mois de juin, ce qui n'est pas aligné sur la stratégie actuelle du PEV de routine. Les participants ont suggéré que pour que cette stratégie soit efficace, la communication et la mobilisation sociale qui accompagnent habituellement une campagne pourraient être assurées parallèlement à la distribution des doses de rappel saisonnières au centre de santé, en tirant parti des bénévoles de la santé communautaire, des parties prenantes et des organisations déjà en place pour contribuer à cette distribution. Cependant, des ressources financières supplémentaires seraient nécessaires, et les partenaires devraient s'engager à soutenir les communications de routine. En outre, certains participants ont suggéré que, pendant les deux premières années précédant la mise en œuvre à grande échelle du RTS,S/AS01$_E$, des CVM pourraient être mis en œuvre dans certaines zones à forte charge de morbidité afin de susciter l'enthousiasme pour le vaccin et d'accroître l'accessibilité à l'introduction.

Tableau 2 : Stratégie d'exécution recommandée par chaque groupe de participants au cours des IDI et de l'atelier national, et justification de la recommandation

| | **Principale stratégie* recommandée et justification de la recommandation** | |
|---|---|---|
| **Responsables de programmes nationaux** | Stratégie 3 : systèmes de distribution basés sur l'âge et mixtes saisonniers | • Les doses de primovaccination dans le système de routine durable et RTS,S améliorent la couverture du PEV<br>• ↑ des doses de rappel saisonniers efficaces avec ↑ couverture des campagnes<br>• Des campagnes pour atteindre ceux qui ont manqué le PEV |
| **Responsables de programmes régionaux et de district** | Stratégie 1 : PEV de routine basé sur l'âge | • S'intègre au système actuel et donc moins cher<br>• Moins de charge pour le système de santé<br>• Moins de dépendance à l'égard des partenaires<br>• Plus durable |
| **Agents de santé** | Stratégie 1 : PEV de routine basé sur l'âge | • Plus facile que le programme existant<br>• Les agents de santé/soignants y sont déjà habitués<br>• Moins cher |
| **Soignants et acteurs communautaires** | Stratégie 3 : systèmes de distribution basés sur l'âge et mixtes saisonniers | • Protection précoce lorsque vous êtes habitués au PEV<br>• Protection saisonnière annuelle par des doses de rappel lorsque les enfants sont plus âgés<br>• Campagnes de porte-à-porte et plus faciles pour les enfants plus âgés |
| **Atelier national*** | Stratégie 4 : PEV de routine basé sur l'âge et la saison | • S'intègre dans le système actuel = moins cher et ↑ durable<br>• ↑ Des doses de rappel saisonnières efficaces |

* Stratégie 4 : le PEV de routine basé sur l'âge et la saison n'a été inclus dans les discussions que lors de l'atelier national, après les IDI. Au cours des IDI, seules les stratégies 1, 2 et 3 ont été envisagées.

**DISCUSSION**

Cette étude a identifié quatre stratégies pour l'administration du RTS,S/$AS01_E$ en même temps que la CPS dans les zones de paludisme saisonnier, définissant la stratégie d'administration comme le calendrier vaccinal du RTS,S/$AS01_E$ et le(s) système(s) de distribution utilisé(s) pour l'administrer. Dans l'ensemble, les participants aux entretiens et à l'atelier national ont préféré le calendrier de vaccination des stratégies 3 et 4, avec les trois premières doses de primovaccination administrées selon un calendrier basé sur l'âge au cours de la première année de vie, et des doses de rappel annuelles saisonnières. Cela s'explique par le fait que, contrairement aux deux autres calendriers de vaccination, les enfants sont à la fois protégés très tôt dans leur vie et reçoivent une protection saisonnière annuelle après l'âge de deux ou trois ans, comme le ferait la stratégie 1 (8). Cependant, la manière dont ces doses de rappel saisonnières devraient être administrées a fait l'objet de

discordances et de discussions, car elles ne s'inscrivent pas dans la stratégie actuelle du PEV, tant en termes de saisonnalité que de groupe d'âge cible.

En dépit de la couverture élevée prévue par les campagnes, les participants à cette étude avaient de grandes inquiétudes quant aux ressources nécessaires et à la charge que l'ajout de CVM annuelles contre le paludisme saisonnier aurait sur le système de santé dans son ensemble. Plusieurs études antérieures ont souligné les effets négatifs que les campagnes de masse peuvent avoir sur les systèmes de santé de routine, en particulier au niveau du district, notamment la motivation financière des agents de santé à travailler sur la campagne plutôt que sur les activités de routine, l'absence d'agents de santé dans les centres de santé et la réduction ou l'interruption des services de routine pendant les campagnes (16–21). Les participants ont insisté sur ce point dans le contexte plus large du Mali, où de nombreuses campagnes de masse sont menées régulièrement, notamment la CPS, la semaine de la nutrition, les médicaments anthelminthiques, la distribution de moustiquaires et les CVM réactives et d'introduction. Bien que l'unique CVM annuelle dans le cadre de la stratégie de systèmes de distribution mixtes en fonction de l'âge et de la saison (stratégie 3) ait été considérée comme plus réalisable que les trois CVM annuelles (stratégie 2), cela se traduirait tout de même par 5 à 6 mois consécutifs de campagnes de lutte contre le paludisme chaque année, ce qui était préoccupant, en particulier pour les participants au niveau des districts. Cette charge de travail associée aux campagnes, en particulier au niveau du district, se reflète dans les résultats, où les responsables de programme de niveau inférieur et les agents de santé ne recommandent pas, dans l'ensemble, les stratégies utilisant les CVM.

En raison des impacts des CVM sur le système de santé, et de la perception de la non-durabilité de ces impacts, ainsi que des coûts élevés et de la nécessité d'un financement par les partenaires, cette étude a révélé un désir général d'intégrer pleinement la fourniture de RTS,S/AS01$_E$ dans le système du PEV de routine. Les participants à tous les niveaux ont estimé que le programme du PEV de routine était un système de distribution plus réalisable et plus durable, déjà connu des communautés et auquel elles faisaient confiance. Cela a conduit à la création d'une quatrième stratégie au cours de l'étude, le PEV de routine basé sur l'âge et les saisons (stratégie 4), avec les trois doses de primovaccination basées sur l'âge et les doses de rappel saisonnières toutes délivrées dans les cliniques du PEV de routine.

Il y avait des inquiétudes quant à la couverture que la stratégie 4 permettrait d'atteindre. Actuellement, tous les vaccins du PEV sont administrés toute l'année dans les cliniques selon un calendrier basé sur l'âge, en vaccinant les enfants jusqu'à 23 mois, le dernier vaccin (MCV-2) étant prévu à l'âge de 15 mois. En revanche, cette stratégie obligerait tous les enfants de moins de cinq ans à se rendre au centre de vaccination du PEV à un moment donné de l'année, avant la saison de transmission du paludisme. Historiquement, les programmes du PEV se sont concentrés sur les enfants de moins de 12 mois, le MCV-2 ayant été récemment introduit comme l'un des premiers vaccins infantiles administrés au-delà de cet âge. Le MCV-2 a connu des couvertures nettement inférieures à celles du MCV-1, en partie à cause de l'idée persistante que le PEV s'arrête après la petite enfance, et de la formation insuffisante des agents de santé, ce qui a entraîné des problèmes d'attitude et de connaissance du PEV au cours de la deuxième année de vie (22,23). La couverture du MCV-2 au Mali est estimée à 33 % (10).

Des interventions de soutien sont nécessaires pour atteindre une couverture élevée de RTS,S/AS01$_E$ au-delà des trois premières doses de primovaccination dans le PEV de routine. Diverses interventions visant à améliorer les vaccinations infantiles de routine ont été testées, y compris des interventions ciblant la communication et la mobilisation, le rappel, les incitations et les stratégies dirigées par les prestataires (24,25). Cependant, si nombre de ces stratégies ont été suggérées pour

améliorer la couverture vaccinale, en particulier au cours de la deuxième année de vie, à notre connaissance, aucune n'a été formellement évaluée ni les contextes dans lesquels elles sont efficaces déterminés (22,23,25–27).

Les participants à cette étude ont suggéré que, dans le contexte malien où le paludisme est très répandu et où la confiance et la demande en vaccins du PEV sont élevées, mais où il n'y a pas d'expérience de la vaccination saisonnière de routine et de la vaccination au-delà de l'âge de 15 mois, les interventions les plus efficaces seraient celles impliquant : des communications et des sensibilisations intensives pour s'assurer que les communautés connaissent et comprennent le nouveau vaccin et la manière de le recevoir ; des rappels aux soignants concernant les doses à venir ; le suivi des enfants qui ne vont pas à l'école ; la formation et la supervision des agents de santé. Une possibilité pour les rappels et le traçage des enfants défaillants serait d'introduire un système de rappel électronique, envoyant des rappels pour les contacts vaccinaux à venir et manqués, qui a montré un certain succès dans de petites études pilotes en Afrique sub-saharienne (28). Dans les zones où le niveau d'alphabétisation est faible, il est possible d'utiliser des SMS vocaux ou des appels téléphoniques (29). Ce système pourrait être associé à un registre électronique de vaccination pour suivre la réception des doses de vaccin au niveau individuel, ce qui permettrait également de surmonter les difficultés prévues dans cette étude en ce qui concerne la conservation des cartes de vaccination et l'évaluation de l'éligibilité aux doses de rappel (30).

Les participants à cette étude ont souligné que les bénévoles de la santé communautaire, qui sont proches des communautés et en qui elles ont confiance, devraient jouer un rôle clé dans la mise en œuvre d'interventions de soutien, notamment en sensibilisant les soignants aux vaccinations à venir et en recherchant et en orientant les enfants qui ne se présentent pas aux vaccinations. Les participants ont noté que si ces travailleurs ont des rôles essentiels, leur participation aux activités de santé de routine a été négligée et sous-financée. Au Kenya, les agents de santé communautaires ont joué un rôle important dans l'augmentation de la couverture vaccinale en recherchant les enfants et en s'assurant qu'ils ne manquent pas ou ne retardent pas leurs vaccinations (31). Les participants ont également suggéré que les distributeurs communautaires de CPS, qui font du porte-à-porte pendant la saison des pluies, pourraient examiner les cartes de vaccination des enfants et transmettre des messages sur le RTS,S/AS01$_E$ et l'orientation vers la clinique du PEV pour les enfants qui n'ont pas reçu leur dose de rappel au cours du mois précédent. L'orientation et les messages sur les RTS,S/AS01$_E$ pendant les contacts de CPS, et vice versa, pourraient faciliter l'intégration des deux programmes et la compréhension de la nécessité des deux interventions. Il sera important de considérer et d'évaluer l'effet que le RTS,S/AS01$_E$ a sur les perceptions et la couverture de CPS, et comment cela est influencé par la manière dont il est administré.

Le principal avantage des CVM discuté par tous les niveaux de cette étude est la forte composante de communication et de mobilisation normalement absente du PEV de routine, qui se traduit par une forte sensibilisation et motivation, et une couverture élevée. Étant donné la nécessité pour les soignants d'amener leurs enfants plus âgés au PEV à un moment précis de l'année dans la stratégie recommandée pour le PEV de routine en fonction de l'âge et des saisons, les participants à l'atelier ont suggéré que la communication et la mobilisation sociale accompagnant habituellement une campagne pourraient être assurées parallèlement à la distribution des doses de rappel saisonnières au centre de santé, assurée par les agents de santé communautaires et d'autres groupes communautaires importants. Cependant, un obstacle à cette démarche serait la volonté des partenaires de soutenir le renforcement des communications et des programmes de routine.

Les participants à cette étude ont également soulevé certains problèmes liés à la distribution des trois doses de primovaccination dans le cadre du programme PEV. On a supposé qu'il était facile

d'intégrer de nouveaux vaccins ou d'autres interventions dans les contacts PEV existants, mais cette étude a soulevé des inquiétudes quant à l'ajout de trois nouvelles doses dans un calendrier PEV de plus en plus chargé. Alors que les soignants de cette étude préféraient souvent que le RTS,S/AS01$_E$ soit administré seul lors des nouveaux contacts, en raison de préoccupations concernant les effets secondaires et la visibilité accrue du vaccin, des défis importants ont été soulevés quant à l'ajout de nouveaux contacts dans le calendrier, et une fois encore, des interventions de soutien axées sur la communication, la mobilisation, les rappels et le rappel ont été suggérées comme étant nécessaires pour que les soignants se rendent à ces nouveaux contacts. En outre, les enseignements tirés de l'étude pilote RTS,S/AS01$_E$ suggèrent que des directives clairement définies et une formation et une supervision solides sont nécessaires pour que les agents de santé puissent mettre en œuvre les nouveaux calendriers vaccinaux, en particulier en ce qui concerne l'éligibilité en fonction de l'âge et ce qui se passe lorsque les enfants ne viennent pas à l'heure prévue (32).

Bien qu'une évaluation réaliste complète n'ait pas été entreprise, la collecte de données qualitatives dans cette étude a permis d'ajouter des approches réalistes au questionnement (6). L'utilisation des configurations de CMO à la fin de l'entretien a été précieuse pour fournir une explication directe et explicite des contextes et mécanismes spécifiques conduisant aux recommandations des participants pour les stratégies de distribution. Par exemple, les agents de santé ont discuté de l'avantage d'être rémunérés pour les campagnes lors des entretiens. Cependant, lorsque cette question a été posée à l'aide d'un CMO, il est apparu clairement que si la compensation motivait les agents de santé dans une certaine mesure, elle ne les incitait pas à distribuer les RTS,S/AS01$_E$ par le biais des CVM en raison de leur charge de travail accrue et de la facilité perçue de la distribution par le PEV. De plus, l'inclusion explicite de questions sur le contexte dans les entretiens a permis de centrer le contexte dans lequel l'administration de RTS,S/AS01$_E$ était envisagée. Par exemple, ces questions ont permis d'établir la perception du succès et de la confiance dans le programme du PEV au Mali, et donc en partie pourquoi les participants considéraient l'introduction du vaccin dans le PEV de routine comme une option plus facile.

Les stratégies d'administration identifiées dans cette étude s'appliquent au-delà du Mali et peuvent être utilisées pour d'autres pays ayant une transmission saisonnière du paludisme. Chaque pays devrait adapter l'administration du vaccin antipaludique à son contexte spécifique, y compris en termes de profil de transmission du paludisme et de saisonnalité, ainsi qu'aux forces de ses systèmes de distribution, en particulier son programme PEV, y compris dans les zones d'insécurité. Lorsque la saisonnalité du paludisme varie, certains pays peuvent choisir de varier la stratégie de distribution utilisée dans le pays. Par exemple, le Ghana fournit actuellement le vaccin RTS,S/AS01$_E$ dans les parties du pays où la transmission est pérenne en utilisant la stratégie 1, mais s'il est étendu dans le pays, il pourrait choisir de fournir des doses de rappel saisonnières du vaccin dans les parties du pays où le paludisme est fortement saisonnier (4,27). De plus, bien que cette étude se soit concentrée sur l'administration du vaccin RTS,S/AS01$_E$, les stratégies de distribution identifiées sont applicables à tout vaccin antipaludique ayant des âges cibles similaires et une efficacité qui diminue avec le temps (nécessitant donc des rappels réguliers), comme le vaccin R21 (33).

Bien que les stratégies de distribution et la définition de ce qu'est une stratégie d'administration du RTS,S/AS01$_E$ avec la CPS soient généralisables au-delà du Mali, les défis et avantages perçus des stratégies et les recommandations faites dans cette étude sont spécifiques au Mali. Cette étude a inclus un large éventail de répondants, mais les gardiens/tuteurs, les agents de santé et les responsables de programme de district n'ont été interrogés que dans deux districts, et donc les perceptions des stratégies de ces groupes étaient spécifiques au contexte de leurs districts. Par exemple, les deux districts sont semi-ruraux, et les gardiens/tuteurs et les agents de santé des

communautés reculées ou urbaines peuvent avoir des perspectives différentes sur les stratégies de distribution. Cependant, l'inclusion des responsables de programmes nationaux et régionaux a permis d'élargir l'applicabilité de ces stratégies. Ils ont soulevé des points plus larges, comme la manière dont les zones d'instabilité et de faiblesse du PEV peuvent affecter le succès des stratégies de distribution. En outre, les responsables de programmes nationaux, régionaux et de district se sont accordés sur les défis et les avantages de chaque stratégie. Une autre limite est que la stratégie 4 n'a été développée qu'après les entretiens, donc bien qu'elle ait été discutée lors de l'atelier national, elle n'a pas été abordée lors des entretiens. Cette étude était également limitée par sa nature prospective, et les recommandations des participants étaient préliminaires et basées sur les données actuellement disponibles au moment de l'étude (novembre 2021 - juillet 2022). Cela ne comprend pas les preuves de l'efficacité relative de chaque stratégie, il est possible que les recommandations des participants diffèrent en présence de telles preuves. Les décisions ultérieures sur la manière d'administrer le vaccin RTS,S/$AS01_E$ au Mali et dans d'autres pays devront prendre en compte d'autres considérations, notamment : l'efficacité modélisée des différentes stratégies d'administration dans des zones d'intensités différentes de paludisme saisonnier ; le rapport coût-efficacité comparatif des stratégies, en particulier pour le choix entre les stratégies 3 et 4 ; le nombre de doses de vaccin nécessaires et disponibles ; et le soutien financier et technique disponible pour les pays.

**CONCLUSIONS**

Quatre stratégies pour l'administration du vaccin RTS,S/$AS01_E$ parallèlement à la CPS dans les pays où la transmission du paludisme est saisonnière ont été présentées. Les considérations clés dans le développement des stratégies d'administration du vaccin contre le paludisme saisonnier ont été soulignées, ainsi que les recommandations pour le Mali où la stratégie préférée était une combinaison de doses de primovaccination basées sur l'âge suivies de doses de rappel saisonnières, toutes administrées par le biais du programme PEV de routine. Des interventions de soutien sont nécessaires pour assurer le succès de la vaccination RTS,S/$AS01_E$, étant donné la nature nouvelle et la complexité de l'administration de doses de vaccin de manière saisonnière et à un groupe d'âge élargi. Il est nécessaire de poursuivre la recherche et l'évaluation de la mise en œuvre de ces nouvelles stratégies, notamment en ce qui concerne les interventions de soutien nécessaires pour en accroître l'efficacité.

**Remerciements**
Les auteurs tiennent à remercier les responsables de programmes, les agents de santé, les gardiens/tuteurs d'enfants et les leaders communautaires qui ont participé à cette étude. Nous tenons également à remercier le PEV et le PNLP au Mali, en particulier le Dr Ibrahima Diarra, Bani Diaby, Aboubacar Traoré, le Dr Idrissa Cissé, le Dr Mamadou H Magassa et Vincent Sanogo, pour leur soutien à cette étude et leurs commentaires sur la conception de l'étude et tout au long de l'étude. Nous remercions également la Direction Régionale de la Santé de Koulikoro et Sikasso et les Districts Sanitaires de Bougouni et de Ouelessebougou pour leur soutien à cette étude. Nous remercions les agents de collecte de données, les transcripteurs et les traducteurs, ainsi que Karen Slater pour son soutien à l'étude. Aucun patient n'a été impliqué dans cette étude.

**Contributions**

JG, HD, JW, ST ont élaboré la première version du protocole de l'étude et du matériel de l'étude, avec le soutien de AD, IS, BG et DC. ST et FK ont réalisé la collecte des données, avec le soutien de

HD. L'analyse des données a été réalisée par JG, ST, JM, HD, JW. JG, JW et HD ont rédigé le manuscrit. Tous les auteurs ont contribué à la révision du manuscrit et ont approuvé la version finale.

**Conflit d'intérêts**
Les auteurs déclarent qu'ils n'ont pas de conflit d'intérêts.

**Financement**
L'étude a été financée par PATH MVI, subvention n° 18269 et le UK Joint Global Health Trials (Department of Health and Social Care, the Foreign, Commonwealth & Development Office, the Global Challenges Research Fund, the Medical Research Council and Wellcome Trust. La bourse financée par le Royaume-Uni fait partie du programme EDCTP2 soutenu par l'Union européenne (subvention n° MR/P006876/1). MR/P006876/1).

**Approbation éthique et consentement à la participation**
L'approbation éthique de l'étude a été obtenue auprès du comité d'éthique de la Faculté de médecine, de pharmacie et d'odontologie de l'Université de Bamako, au Mali, et du comité d'éthique de la London School of Hygiene and Tropical Medicine, au Royaume-Uni. Tous les participants à l'entretien ont donné leur consentement éclairé par écrit.

**Déclaration sur la disponibilité des données**
Les parties des ensembles de données utilisés et/ou analysés sont disponibles auprès de l'auteur correspondant sur demande raisonnable. Les fichiers de transcription des responsables de programme ne seront pas partagés dans leur intégralité car les participants sont potentiellement identifiables par une personne familière du contexte en raison de la description de leurs rôles au sein des programmes de vaccination et de lutte contre le paludisme, et de la manière dont leurs rôles influencent leurs réponses sur l'administration du vaccin.

**References**

1. ACCESS-SMC Partnership. Effectiveness of seasonal malaria chemoprevention at scale in west and central Africa: an observational study. The Lancet. 2020;396(10265):1829–40.
2. World Health Organization. World malaria report 2021. Geneva; 2021.
3. Chandramohan D, Dicko A, Zongo I, et al. Effect of Adding Azithromycin to Seasonal Malaria Chemoprevention. New England Journal of Medicine. 2019;380(23):2197–206.
4. World Health Organization. Full Evidence Report on the RTS,S/AS01 Malaria Vaccine. 2021.
5. World Health Organization. Malaria: The malaria vaccine implementation programme (MVIP) [Internet]. 2020 [cited 2022 Dec 2]. Available from: https://www.who.int/news-room/questions-and-answers/item/malaria-vaccine-implementation-programme
6. Manzano A. The craft of interviewing in realist evaluation. Evaluation. 2016;22(3):342–60.

7. Pawson R, Tilley N. Realistic Evaluation. London: SAGE Publications Ltd; 1997.

8. Dicko A, Ouedraogo JB, Zongo I, et al. Protection against seasonal malaria for five years with vaccination and chemoprevention. 2023 [Unpublished manuscript].

9. Chandramohan D, Zongo I, Sagara I, et al. Seasonal Malaria Vaccination with or without Seasonal Malaria Chemoprevention. N Engl J Med. 2021;385:1005–1017.

10. World Health Organization. WHO Immunization Data portal- Mali [Internet]. [cited 2022 Dec 2]. Available from: https://immunizationdata.who.int/pages/profiles/mli.html

11. de Silva MJ, Breuer E, Lee L, et al. Theory of Change: A theory-driven approach to enhance the Medical Research Council's framework for complex interventions. Trials. 2014;15(1):1–13.

12. World Health Organization. Everybody's business – strengthening health systems to improve health outcomes: WHO's framework for action. 2007.

13. Rogers E. Diffusion of innovations. 5th ed. New York: Free Press; 2005.

14. Bowen DJ, Kreuter M, Spring B, et al. How We Design Feasibility Studies. Vol. American Journal of Preventive Medicine. 2009;36:452–7.

15. Gale NK, Heath G, Cameron E, et al. Using the framework method for the analysis of qualitative data in multi-disciplinary health research. BMC Med Res Methodol. 2013;13(1):1–8.

16. Dietz V, Cutts F. The Use of Mass Campaigns in the Expanded Program on Immunization: A Review of Reported Advantages and Disadvantages. Int J Health Serv. 1997;27(4):767–90.

17. Chakrabarti A, Grépin KA, Helleringer S. The impact of supplementary immunization activities on routine vaccination coverage: An instrumental variable analysis in five low-income countries. PLoS One. 2019;14(2).

18. Cavalli A, Bamba SI, Traore MN, et al. Interactions between Global Health Initiatives and country health systems: the case of a neglected tropical diseases control program in Mali. PLoS Negl Trop Dis. 2010;4(8).

19. Mounier-Jack S, Edengue JM, Lagarde M, et al. One year of campaigns in Cameroon: effects on routine health services. Health Policy Plan. 2016;31(9):1225.

20. Mounier-Jack S, Burchett HED, Griffiths UK, et al. Meningococcal vaccine introduction in Mali through mass campaigns and its impact on the health system. Glob Health Sci Pract. 2014;2(1):117–29.

21. Coulibaly Y, Cavalli A, van Dormael M, et al. Programme activities: a major burden for district health systems? Trop Med & Int Health. 2008;13(12):1430–2.

22. Masresha B, Luce R, Okeibunor J, et al. Introduction of The Second Dose of Measles Containing Vaccine in The Childhood Vaccination Programs Within The WHO Africa Region -Lessons Learnt. J Immunol Sci. 2018;2:113–21.

23. World Health Organization. Establishing and strengthening immunization in the second year of life Practices for vaccination beyond infancy. 2018.

24. Oyo-Ita A, Wiysonge CS, Oringanje C, et al. Interventions for improving coverage of childhood immunisation in low- and middle-income countries. Cochrane Database of Syst Rev. 2016;7(7).

25. Omoniyi OS, Williams I. Realist synthesis of the international theory and evidence on strategies to improve childhood vaccination in low-and middle-income countries: Developing strategies for the nigerian healthcare system. Int J Health Policy Manag. 2020;9(7):274–85.

26. Nyaku M, Wardle M, Eng J vanden, et al. Immunization delivery in the second year of life in Ghana: the need for a multi-faceted approach. Pan Afr Med J. 2017;27:4.

27. Grant J, Gyan T, Agbokey F, et al. Challenges and lessons learned during the planning and early implementation of the RTS,S/AS01E malaria vaccine in three regions of Ghana: a qualitative study. Malar J. 2022;21(1).

28. Eze P, Lawani LO, Acharya Y. Short message service (SMS) reminders for childhood immunisation in low-income and middle-income countries: A systematic review and meta-analysis. BMJ Global Health. 2021;6(7).

29. Diallo O, Schlumberger M, Sanou C, et al. Recours aux SMS pour convoquer les mères aux séances de vaccination à Bobo-Dioulasso. Bulletin de la Societe de Pathologie Exotique. 2012;105(4):291–5.

30. Namageyo-Funa A, Samuel A, Bloland P, et al. Considerations for the development and implementation of electronic immunization registries in africa. Pan Afr Med J. 2018;30.

31. Nzioki JM, Ouma J, Ombaka JH, et al. Community health worker interventions are key to optimal infant immunization coverage, evidence from a pretest-posttest experiment in Mwingi, Kenya. Pan Afr M J. 2017;28.

32. World Health Organization. Learning lessons from the pilots: overcoming knowledge gaps around the malaria vaccine schedule in support of vaccine uptake [Internet]. 2022 [cited 2023 Mar 13]. Available from: https://www.who.int/news-room/feature-stories/detail/learning-lessons-from-the-pilots--overcoming-knowledge-gaps-around-the-malaria-vaccine-schedule-in-support-of-vaccine-uptake

33. Datoo MS, Natama HM, Somé A, et al. Efficacy and immunogenicity of R21/Matrix-M vaccine against clinical malaria after 2 years' follow-up in children in Burkina Faso: a phase 1/2b randomised controlled trial. Lancet Infect Dis. 2022;22(12):1728–36.
